# Synaptic Plasticity—Intrinsic Excitability and Antidepressant Discovery

**DOI:** 10.3390/biomedicines14061265

**Published:** 2026-06-01

**Authors:** Masaru Tanaka

**Affiliations:** HUN-REN-SZTE Neuroscience Research Group, Danube Neuroscience Research Laboratory, Hungarian Research Network, University of Szeged (HUN-REN-SZTE), 6725 Szeged, Hungary; tanaka.masaru.1@med.u-szeged.hu

**Keywords:** synaptic plasticity, neuronal excitability, ketamine, esketamine, neurogenesis, signal transduction, excitatory postsynaptic potentials, α-amino-3-hydroxy-5-methyl-4-isoxazolepropionic acid (AMPA) receptors, antidepressive agents, major depressive disorder (MDD)

## Abstract

Major depressive disorder remains a leading cause of disability, and decades of monoamine-centered pharmacology have yielded delayed and often incomplete relief. Rapid-acting antidepressants reshaped the field by linking swift symptom improvement to glutamatergic plasticity, yet durable benefit depends on how newly reconfigured circuits are stabilized and tuned. This review synthesizes evidence that antidepressant efficacy arises from the coordinated engagement of synaptic plasticity, spanning induction and consolidation, and intrinsic excitability, which provides gain control, and proposes an integrated framework to guide future discovery. It first outlines induction through N-methyl-D-aspartate receptors (NMDARs) and α-amino-3-hydroxy-5-methyl-4-isoxazolepropionic acid receptors (AMPARs), exemplified by ketamine and esketamine, followed by consolidation mediated by tropomyosin receptor kinase B (TrkB) signaling, translational disinhibition via eukaryotic elongation factor 2 kinase (eEF2K), and presynaptic stabilization indexed by synaptic vesicle glycoprotein 2A (SV2A); together, these processes transform transient potentiation into persistent network change. It then highlights intrinsic excitability, emphasizing voltage-gated potassium channel subfamily Q (Kv7), hyperpolarization-activated cyclic nucleotide-gated (HCN), and G protein-gated inwardly rectifying potassium (GIRK) channels as circuit-level governors that normalize firing and limit relapse-prone hyperexcitability. Finally, it presents the Induction–Consolidation–Maintenance (ICM) framework as a hypothesis-generating roadmap for future studies, with SV2A positron emission tomography (PET), electroencephalography (EEG), and functional magnetic resonance imaging (fMRI) biomarkers discussed as candidate tools rather than validated guides for treatment timing or patient selection. The proposed contribution is not another list of plasticity pathways, but a phase-specific model that links synaptic induction, consolidation, and excitability-based maintenance to distinct therapeutic windows, biomarkers, and relapse-prevention strategies.

## 1. Introduction: Beyond Monoamines to Plasticity and Excitability

Depression remains one of the leading causes of disability worldwide, with prevalence and disability adjusted life years increasing steadily over the past three decades and accelerating after 2019 [1,2,3]. Nearly one in five individuals experience clinically significant depressive symptoms across the lifespan, with onset often occurring in childhood or adolescence [4,5,6]. These trends carry profound consequences for education, productivity, physical health, and health care systems, underscoring depression as a sustained and escalating global burden rather than a transient public health challenge [1,6,7].

For much of the modern era, the monoamine hypothesis has provided a unifying framework for understanding depression and guiding antidepressant discovery [7,8,9,10]. By linking symptoms to deficiencies in serotonin, norepinephrine, and dopamine signaling, this model delivered conceptual clarity and enabled the development of multiple effective treatments [7,8,9]. However, its clinical limitations have become increasingly apparent [7,8]. Many patients fail to achieve remission, a substantial proportion develop treatment-resistant depression, and relapse rates remain high even after apparent recovery [7,9,11]. Therapeutic benefits typically emerge only after weeks of treatment, a delay that complicates acute care and is difficult to reconcile with the rapid pharmacological effects of monoaminergic drugs [7,8,11,12].

Compounding these challenges, depression is highly heterogeneous [7,11,13,14]. Genetic, transcriptomic, and neurobiological studies point to diverse alterations in immune signaling, synaptic plasticity, and network function across patient subgroups, arguing against a single deficit model [7]. Together, these observations motivate a mechanistic expansion beyond neurotransmitter availability toward synaptic plasticity, intrinsic excitability, and circuit-level regulation as core drivers of antidepressant response and discovery [7,10,11,13,15,16]. This expansion should not be read as a replacement for other disease mechanisms. Neuroimmune signaling, astrocyte function, metabolic state, endocrine regulation, sex-specific biology, and developmental timing interact with plasticity and excitability, shaping both vulnerability and treatment response. Recent evidence from septic shock extends this systems-level view by showing that acute physiological instability can leave measurable neuropsychiatric signatures. Reduced cardiac index, low mean arterial pressure, and elevated lactate were associated with delirium, depression, anxiety, and post-traumatic stress symptoms, suggesting that mood and cognitive outcomes may function as clinically meaningful readouts of disrupted perfusion, inflammation, and brain network vulnerability [17].

The discovery of ketamine marked a conceptual turning point in antidepressant research by demonstrating that depressive symptoms can improve within hours rather than weeks [18,19,20]. Unlike traditional agents, ketamine exerts its effects through glutamatergic modulation and rapid neuroplastic change, challenging the assumption that therapeutic benefit must arise from slow monoaminergic adaptation [18,21,22]. Ketamine is introduced here as the clinical entry point into rapid plasticity-based antidepressant action. Detailed molecular steps involving NMDAR disinhibition, AMPAR throughput, brain-derived neurotrophic factor (BDNF)-TrkB signaling, and mTOR activation are discussed in Section 2.1.

Subsequent work has reinforced this plasticity framework. Ketamine metabolites and enantiomers exhibit synaptogenic properties with distinct side-effect profiles, while downstream signaling pathways such as extracellular signal-regulated kinase (ERK) and metaplasticity mechanisms shape the persistence and scalability of plastic change [23]. Importantly, ketamine can restore homeostatic synaptic balance and normalize dopamine-dependent plasticity without disrupting learning-related potentiation, indicating broad circuit-level repair rather than nonspecific excitation [21]. These mechanisms stand in sharp contrast to monoaminergic antidepressants, which rely on chronic receptor engagement and gradual transcriptional remodeling with delayed and variable clinical outcomes [18]. As a result, the field has shifted toward synaptic remodeling, intrinsic excitability, and plasticity consolidation as central therapeutic targets, redefining how antidepressant efficacy is conceptualized and pursued [10,18,20].

Intrinsic excitability describes a neuron’s inherent tendency to fire action potentials in response to input, determined by its repertoire of voltage and ligand-gated ion channels rather than by changes in synaptic strength [24,25,26]. Unlike synaptic plasticity, which modifies the efficacy of connections between neurons, intrinsic excitability regulates neuronal gain and input–output transformations at the single cell level [24,25,26]. In prefrontal and hippocampal circuits, channels such as voltage-gated potassium channel subfamily Q (Kv7), hyperpolarization-activated cyclic nucleotide-gated channel (HCN), and G protein-gated inwardly rectifying potassium gated channel (GIRK; Kir3.x) serve as key determinants of firing probability and network stability [27,28,29]. Kv7 or M channels act as powerful brakes on depolarization, with their inhibition increasing pyramidal neuron excitability and their activation dampening gain. HCN channels shape resting conductance, resonance, and temporal integration, linking altered channel function to stress sensitivity and anhedonic phenotypes [27,28,30]. GIRK channels further stabilize membrane potential and constrain excitability, contributing to mood regulation and cognitive control [30,31].

Stress robustly engages these mechanisms [27,28,29,30]. Chronic stress induces cell-type-specific shifts in intrinsic excitability within medial prefrontal and hippocampal neurons, biasing circuits toward vulnerability or resilience depending on channel composition and neuromodulatory state [25,26]. These changes can occur independently of synaptic remodeling yet powerfully influence circuit output and behavioral state [24,26]. Despite their central role in regulating mood relevant networks, intrinsic excitability mechanisms remain underexplored pharmacologically [30,31]. With few exceptions, systematic targeting of Kv7, HCN, or GIRK channels in depression has lagged behind synaptic plasticity-based approaches, representing a major and underdeveloped opportunity for antidepressant discovery [12,27,30,31].

This review advances a dual framework in which synaptic plasticity and intrinsic excitability jointly govern depressive pathophysiology and antidepressant response. Evidence from rapid-acting interventions demonstrates that restoring synaptic remodeling can rapidly realign network function from molecular signaling to behavior, with strong translational continuity from animal models to human studies. Yet synaptic plasticity alone is insufficient. Dynamic regulation of intrinsic excitability, through ion channel dependent control of neuronal gain and firing probability, acts in parallel to shape circuit output, resilience, and vulnerability [32,33]. The novelty of the proposed ICM framework lies in treating antidepressant response as a temporally ordered control problem rather than as a single plasticity event. Whereas prior neuroplasticity-centered models have emphasized synaptogenesis, trophic signaling, or glutamatergic induction, the present framework adds intrinsic excitability as a gain-control layer that determines whether newly remodeled circuits remain stable, adaptive, and relapse resistant. This integration links synaptic remodeling to ion channel dependent control of firing gain, circuit stability, and relapse vulnerability. This structure may help future studies align induction, consolidation, and maintenance with candidate biomarkers, targets, and treatment windows. These clinical links should be interpreted as future-facing hypotheses rather than established precision-medicine tools, because reproducible stratification, validated biomarkers, and durable relapse-prevention protocols remain incomplete. The conceptual contribution of this review is therefore threefold. First, it separates rapid antidepressant plasticity into mechanistically distinct induction, consolidation, and maintenance phases. Second, it positions intrinsic excitability as the maintenance layer that determines whether remodeled synapses remain adaptive or relapse prone. Third, it converts these phases into a translational roadmap that links targets, biomarkers, timing, and clinical phenotypes. Accordingly, the ICM framework is intended as an organizing scaffold rather than a complete causal theory of depression. Its value lies in mapping one tractable dimension of antidepressant action while leaving room for immune, glial, metabolic, endocrine, sex-related, and developmental modifiers. Because this is a narrative review, the framework is used to organize heterogeneous evidence rather than to rank mechanisms by clinical readiness. Ketamine is treated as the best-established entry point into rapid plasticity, while non-ketamine pathways are discussed according to their current evidentiary maturity.

## 2. Induction and Consolidation of Synaptic Plasticity

Synaptic plasticity comprises two interdependent phases: induction, in which patterns of neuronal activity rapidly alter synaptic efficacy, and consolidation, in which these initially labile changes are stabilized to support persistent circuit reorganization [34,35]. This distinction is particularly relevant to contemporary models of antidepressant action, which increasingly place adaptive plasticity, rather than monoaminergic correction alone, at the center of therapeutic response [36,37]. Within this framework, glutamate serves as the principal excitatory neurotransmitter and the primary molecular interface between acute cellular activation and longer-term synaptic remodeling [38,39]. However, glutamate-dependent induction represents only the opening stage of the process [38,40]. For early plasticity to last, downstream signaling must stabilize structure, transcription, and protein synthesis [34,41]. These events appear to unfold in a coordinated sequence. Glutamatergic signaling first initiates rapid synaptic potentiation and plasticity-related spine dynamics; tropomyosin receptor kinase B (TrkB) activation then supports the transition from transient potentiation to stabilized synaptic reinforcement; and translational control mechanisms determine whether these newly engaged synapses acquire the molecular substrates required for persistence [36,41]. Together, these interacting cascades provide a mechanistic framework for understanding how brief pharmacological perturbations can drive durable antidepressant effects. The following subsections examine this sequence in turn, focusing on glutamate-mediated plasticity drivers, TrkB-dependent consolidation mechanisms, and translational regulation through eukaryotic elongation factor 2 kinase (eEF2K)-related pathways. This organization is not intended to reintroduce established ketamine, BDNF-TrkB, or AMPAR mechanisms as isolated pathways. Instead, these mechanisms are used as phase markers within the ICM framework: glutamatergic signaling defines induction, TrkB and translational control define consolidation, and presynaptic and excitability mechanisms define maintenance.

### 2.1. Glutamate Plasticity Drivers

Ketamine marks a paradigm shift in antidepressant pharmacology by repositioning glutamatergic plasticity as the primary therapeutic mechanism rather than a downstream adaptation [20,42]. At subanesthetic doses, ketamine preferentially antagonizes N-methyl-D-aspartate receptors (NMDARs) on fast spiking GABAergic interneurons, releasing pyramidal neurons from inhibitory control [42,43,44,45,46]. The result is a brief but robust glutamate surge that drives enhanced α-amino-3-hydroxy-5-methyl-4-isoxazolepropionic acid receptor (AMPAR) throughput and calcium influx, including through calcium permeable AMPA channels [20,42,47]. This AMPA dominated signaling window is the critical gate for rapid synaptic change [20,42,48]. Elevated postsynaptic activity triggers BDNF release and TrkB receptor activation, which in turn engages mechanistic target of rapamycin complex 1 (mTORC1) dependent translational programs supporting dendritic spine growth and synaptic strengthening [20,42,48,49,50]. In parallel, calcium/calmodulin-dependent protein kinase II (CaMKII) acts as a rapid activity sensor, coordinating phosphorylation events and structural stabilization at newly potentiated synapses. These cascades converge to produce long-term potentiation (LTP) like synaptic potentiation across corticolimbic circuits within hours, mirroring the temporal profile of ketamine’s antidepressant effects [20,33,48]. Importantly, this mechanism reframes NMDAR antagonism as permissive rather than suppressive, with intact upstream NMDA signaling required to enable AMPA driven plasticity [20,44,45,51]. By inducing rapid, experience independent synaptic remodeling, ketamine redefines both the mechanism and timeline of antidepressant action [20,33,42]. This shifts the therapeutic focus from slow neuromodulatory correction to rapid circuit remodeling [20,47,48]. This subsection therefore serves as the main mechanistic account of ketamine linked induction, while later sections focus on consolidation, maintenance, or translational implications. This emphasis reflects the depth of clinical and mechanistic evidence for ketamine and esketamine, not an assumption that ketamine mechanisms exhaust antidepressant biology. Even this induction sequence is not strictly linear. Glutamatergic signaling is shaped by astrocytic glutamate handling, inflammatory tone, energy availability, hormonal state, and prior developmental or stress exposure.

Esketamine and dextromethorphan (DXM)-bupropion converge on glutamate-driven plasticity but diverge in how they bias circuit entry and clinical expression [52,53,54]. Esketamine provides a more NMDAR-centered entry into the induction pathway described above, producing rapid clinical effects but also transient dissociation and sympathomimetic effects [45,46,47,49,52,55,56]. By contrast, DXM-bupropion operates through a broader multimodal profile. Weak NMDA antagonism is complemented by sigma 1 receptor activation and dopamine transporter inhibition, shaping excitability, stress resilience, and neurotrophic tone alongside glutamatergic mechanisms [57,58,59]. Clinically, esketamine nasal spray demonstrates rapid efficacy in treatment-resistant depression (TRD), with improvements emerging within hours to days and sustained by maintenance dosing [53]. Common adverse effects include dissociation, dizziness, and blood pressure (BP) elevation [52,53,55]. Perioperative studies in cesarean delivery extend this risk–benefit profile beyond treatment-resistant depression, suggesting that low-dose esketamine may reduce post-cesarean pain and postpartum depressive symptoms while still requiring caution because of neurological and hemodynamic adverse effects [60]. This context is clinically useful because it frames esketamine as both an analgesic and rapid-acting antidepressant candidate, but not yet as a routine perioperative intervention without larger, standardized trials. DXM-bupropion shows early and durable symptom reduction in phase 3 trials, including GEMINI (Global Evaluation of the Efficacy and Safety of AXS-05 [DXM–bupropion] in Major Depressive Disorder), with significant Montgomery–Åsberg Depression Rating Scale (MADRS) improvements and minimal psychotomimetic liability [55,61,62]. Its oral administration and milder side-effect profile improve accessibility [52,63]. Mechanistic overlap lies in rapid plasticity induction, while divergence reflects dopaminergic and sigma mediated modulation in DXM bupropion versus the NMDA centric and dissociative signature of esketamine [52,54,64]. Preclinical studies broadly support this induction logic: ketamine and related glutamatergic interventions restore stress-sensitive dendritic structure, synaptic protein expression, and behavioral readouts, with BDNF-mTOR signaling serving as a recurrent but not exclusive molecular hub [46,65,66,67,68,69,70,71,72,73,74,75].

This plasticity program is not ketamine specific. Other glutamate-biased or plasticity-promoting interventions produce overlapping synaptic and behavioral effects, supporting shared pathway engagement while leaving compound-specific mechanisms, regional effects, and durability unresolved [65,66,67,68,73,75,76,77,78,79,80]. Rapid glutamate targeting strategies deliver antidepressant effects that are often short lived [22,81,82]. Across clinical trials, ketamine and esketamine reliably produce rapid symptom relief, yet responses frequently decay within days to weeks, with high relapse rates following discontinuation [81,82,83]. Maintenance regimens extend benefit for some patients but only partially offset this erosion [82,83,84]. Dissociative and psychotomimetic effects are common, typically transient, and sometimes accompanied by cardiovascular changes [85,86,87]. Whether dissociation contributes to efficacy remains unclear, complicating risk-benefit assessment and patient acceptability [85,88,89]. Longer term safety signals are still emerging, including concerns related to abuse liability, urinary symptoms, and cumulative neurocognitive effects with repeated exposure [82,86,90]. Mechanistic uncertainty further constrains translation. The relative roles of synaptic versus extrasynaptic NMDARs, interactions with parallel neurotransmitter systems, and optimal dose response relationships remain incompletely defined [20,48,64]. Clinical response is highly heterogeneous, and robust predictors are lacking, underscoring the need for biomarkers and stratified treatment strategies [85,91,92,93]. Equally important, biomarker findings across ketamine studies remain inconsistent. Changes in glutamate metabolites, EEG spectral indices, inflammatory markers, BDNF, and network connectivity have been reported, but effect directions, timing, and predictive value vary across cohorts and protocols [85,94,95]. These discrepancies caution against treating any single biomarker as a validated response guide. Together with limited durability, relapse after discontinuation, dissociation, blood pressure elevation, abuse liability, and uncertain long-term safety, these biomarker limitations constrain the near-term clinical reach of rapid glutamatergic strategies.

**Figure 1:**
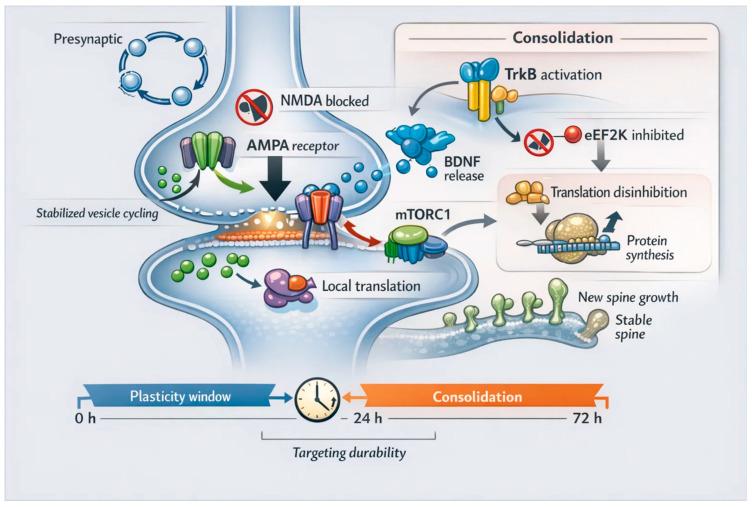
Plasticity window and consolidation timeline in rapid antidepressant action. Rapid-acting antidepressant effects can be framed as a staged transition from induction to consolidation. During induction, NMDAR antagonism, often through interneuron disinhibition, increases glutamate release and AMPAR throughput, opening a brief plasticity window over minutes to hours. Early BDNF release and mTORC1 activation support synaptic remodeling and dendritic spine formation, but these changes remain labile. Durable benefit requires consolidation over hours to days, during which TrkB signaling stabilizes potentiated synapses, eEF2K inhibition sustains local protein synthesis, and SV2A supports presynaptic vesicle cycling. The key concept is timing: adjunctive interventions may be most effective when aligned with the consolidation phase. AMPAR, α-amino-3-hydroxy-5-methyl-4-isoxazolepropionic acid receptor; BDNF, brain-derived neurotrophic factor; eEF2K, eukaryotic elongation factor 2 kinase; mTORC1, mechanistic target of rapamycin complex 1; NMDAR, N-methyl-D-aspartate receptor; SV2A, synaptic vesicle glycoprotein 2A; TrkB, tropomyosin receptor kinase B.

Next-generation glutamate therapies aim to preserve rapid antidepressant plasticity while minimizing dissociative and psychotomimetic effects [96,97]. One avenue centers on AMPAR facilitation [97,98,99]. Classical ampakines and newer agents such as TAK-653 and LT-102 enhance synaptic gain, strengthen BDNF signaling, and engage CaMKII, cyclic adenosine monophosphate (cAMP) response element–binding protein (CREB), protein kinase B (AKT), and mTOR pathways, producing antidepressant-like effects with favorable tolerability [98,100]. Related compounds, including tianeptine and selected iridoids, similarly promote AMPAR trafficking and mTOR-linked plasticity, supporting sustained circuit remodeling [97,99,101]. These approaches position AMPA throughput as a proximal and potentially safer driver of durable synaptic efficacy [98,99]. A complementary strategy involves partial NMDAR modulation, but this pathway illustrates the gap between promising early biology and later clinical translation [96,97]. Rapastinel and related glycine-site modulators were designed to facilitate plasticity without dissociation, yet rapastinel failed to meet primary endpoints in three pivotal phase 3 adjunctive MDD trials, and a fourth study was considered unlikely to meet primary and key secondary endpoints [102,103,104]. Thus, partial NMDAR modulation should be presented as an instructive translational failure as well as a mechanistic strategy, emphasizing the need for replication, dose-response clarity, and clinically meaningful endpoints [102,104]. This example emphasizes that mechanistic promise in glutamatergic modulation does not necessarily predict clinical success [52,96,97]. It also highlights the need for replication, clinically meaningful endpoints, and clearer dose-response relationships before next-generation glutamatergic modulators are considered translationally mature. Figure 1 is intended as a conceptual timing scaffold rather than a complete signaling map; heterogeneous upstream modifiers, regional differences, failed candidates, and safety constraints are discussed in the accompanying text.

### 2.2. TrkB PAMs/Direct Agonists

The BDNF–TrkB axis functions as a master regulator that converts transient plasticity into stable synaptic reinforcement [105,106]. At the level of individual spines, activity-dependent BDNF release activates TrkB in an autocrine loop that is essential for both structural and functional long-term potentiation [105,107,108]. This signaling captures upstream NMDAR and CaMKII activity and stabilizes it locally, allowing newly formed or enlarged spines to persist beyond the induction phase [107,108,109]. Within microcircuits, TrkB signaling implements a refinement rule in which mature BDNF strengthens coactive synapses while precursor BDNF (proBDNF) weakens poorly correlated inputs, sharpening ensembles that encode shared activity patterns [105,110,111]. This balance is dynamically regulated by extracellular proteolytic conversion of proBDNF to mature BDNF during coordinated firing [105,110,111].

Local consolidation is coupled to long-range stabilization through TrkB signaling endosomes that traffic to the soma and engage CREB and mTOR-dependent transcription [106,109,112]. These programs align gene expression, dendritic protein synthesis, and cytoskeletal remodeling to support late-phase potentiation and network persistence [106,112,113]. Through coordinated control of spine stability, transcription, and local translation, TrkB provides the molecular bridge that locks labile plasticity into enduring circuit change [105,112,113]. Preclinical studies identify TrkB positive allosteric modulators as stabilizers of induced plasticity, converting transient synaptic change into a more durable circuit state [114,115]. Preclinical studies position TrkB positive allosteric modulators as consolidation-focused tools that may prolong induced plasticity by stabilizing activity-dependent synaptic changes, rather than by reinitiating the full ketamine-linked induction cascade [33,48,76,116,117,118,119,120].

TrkB signaling further refines network architecture by stabilizing coactive synaptic clusters while pruning asynchronous inputs [116,117]. Endosomal TrkB signaling propagates to the soma, engaging CREB and mTOR pathways that link dendritic demands to transcriptional output [76,119,121]. Across disease models, TrkB-positive allosteric modulators (PAMs) rescue spine density and preserve mature spine morphology, supporting a model in which TrkB modulation extends antidepressant like plasticity through sustained structural and proteomic reinforcement [33,116,118]. Clinical translation of TrkB targeting strategies aims to harness neurotrophic efficacy while bypassing the limitations of direct BDNF delivery [122,123,124]. Recombinant BDNF performs poorly in humans due to rapid degradation, limited brain penetration, and unstable exposure profiles [122,123,125]. Its inability to efficiently cross the blood-brain barrier (BBB) and the risk of off-target p75 neurotrophin receptor (p75NTR) activation further constrain therapeutic use [122,125,126]. Small molecule TrkB agonists and positive allosteric modulators provide a more tractable route [124,127,128]. Compounds derived from 7,8-DHF show oral bioavailability and central TrkB engagement, with prodrug formulations improving brain exposure and pharmacokinetics [127,128,129]. Optimized analogs demonstrate sustained signaling with acceptable safety during repeated dosing in preclinical models. Other candidates report high brain to plasma ratios, selective TrkB activation, and low peripheral toxicity, alongside improvements in synaptic structure and cognition [127,129]. Early pharmacokinetic and safety data suggest feasibility, though human data remain sparse [122,127,130]. Nevertheless, the translational status of TrkB potentiation remains early. Several proposed low molecular weight TrkB agonists have raised reproducibility and target specificity concerns, and central exposure remains difficult to optimize across scaffolds [131,132]. Therefore, TrkB PAMs should be framed as experimental consolidation tools rather than near-term antidepressant candidates. Biologic approaches such as agonistic antibodies and peptide-based TrkB activators extend half-life and avoid p75NTR engagement but face delivery challenges [123,124,133]. Ongoing debate over target specificity highlights the need for rigorous validation and BBB informed medicinal chemistry [122,123,124]. These concerns also complicate interpretation of positive preclinical findings. Apparent TrkB activation can depend on assay context, compound purity, pathway bias, and indirect network effects, making replication across laboratories and orthogonal target-engagement assays essential before antidepressant relevance is inferred [131,132]. Thus, TrkB modulators should be interpreted as mechanistically plausible consolidation tools rather than clinically established antidepressant strategies.

Clinical translation of TrkB modulation faces three tightly coupled challenges. Receptor specificity remains the first bottleneck [132,134]. Many proposed small molecules show inconsistent TrkB engagement, partial agonism, or off-target activity across kinome and G protein–coupled receptor (GPCR) panels, with signaling outputs that only partially resemble endogenous BDNF [131,132,134]. This raises concerns about reproducibility, pathway bias, and unintended network effects [131,132]. A second issue is theoretical oncogenicity. Sustained TrkB activation enhances survival and growth signaling, and Trk family receptors are established oncogenic drivers when aberrantly engaged [135,136,137]. Chronic exposure could lower apoptotic thresholds or promote maladaptive persistence, arguing for dose ceilings, intermittent schedules, and careful long term surveillance [135,136,137]. This concern should be viewed as part of a broader safety problem, because prolonged amplification of plasticity pathways may stabilize maladaptive ensembles as well as adaptive synapses if timing, dosing, or regional engagement are poorly controlled. Third, pharmacokinetics remains limiting. Many scaffolds suffer rapid clearance, metabolic instability, and uneven brain exposure [62,131,132]. Prodrug strategies help but durability and regional concentration remain difficult to control [138,139,140]. Despite these hurdles, prospects are improving [138,139,140]. Replace with: Next generation TrkB-PAM scaffolds emphasize biased and bitopic allostery to improve pathway selectivity and reduce off-target signaling [140]. Brain selective prodrugs, circuit-targeted delivery, and refined medicinal chemistry offer routes to safer and more precise neurotrophic reinforcement [138,139,140].

### 2.3. eEF2K Inhibitors

Translational control is a central driver of rapid synaptic plasticity, with eEF2K acting as a molecular brake on protein synthesis [141,142,143]. Under resting conditions, eEF2K phosphorylates eEF2 and slows ribosomal elongation, constraining dendritic translation [141,142,144]. This restraint is maintained by tonic NMDAR signaling [141,143]. When NMDAR activity is reduced, as with ketamine or (2R,6R)-hydroxynorketamine (2R,6R-HNK), eEF2K activity falls, eEF2 becomes dephosphorylated, and translational inhibition is rapidly lifted [141,143,145]. Protein synthesis accelerates within minutes [142,143,145]. Among the earliest products is BDNF, linking eEF2K suppression to the consolidation machinery described above [141,145]. Experimental inhibition or genetic reduction of eEF2K similarly facilitates synaptic strengthening and LTP [141,144,145]. Electrophysiological signatures include larger miniature excitatory postsynaptic currents (EPSCs) and rapid AMPAR insertion [141,144,145]. Behavioral assays parallel these synaptic changes, showing fast antidepressant like responses across multiple paradigms [143,144,145]. Together, these findings position eEF2K as a critical link between NMDA antagonism, translational disinhibition, and rapid mood relevant plasticity.

Preclinical studies show that blocking eEF2K reproduces the core synaptic and behavioral effects of ketamine [146,147,148]. Acute pharmacologic or activity-dependent inhibition of eEF2K rapidly lifts elongation control, triggering a burst of dendritic protein synthesis [146,147,148]. This response prominently includes BDNF, which is required for downstream synaptic potentiation and antidepressant like behavior [74,146,148]. At the synaptic level, eEF2K inhibition increases AMPAR-mediated miniature EPSC amplitudes and induces rapid synaptic scaling, closely matching the profile seen after ketamine or 2R,6R-HNK [146,149,150]. Electrophysiological recordings reveal strengthened hippocampal transmission, facilitated long term potentiation, and fast AMPAR insertion, all of which collapse under AMPA blockade [146,147,149]. Behavioral readouts align with these cellular effects [146,147,148]. Rodents show reduced immobility in forced swim and tail suspension tests, improved novelty suppressed feeding, and reversal of stress-induced anhedonia [146,147,151]. Disruption of BDNF signaling abolishes these gains, while direct eEF2K inhibition is sufficient to restore synaptic efficacy and produce rapid antidepressant-like outcomes [74,148].

eEF2K inhibition offers a distinctive advantage by extending plasticity without driving excitotoxicity [152,153,154]. Rather than increasing synapse number or global firing, it selectively scales AMPAR mediated currents, producing a controlled gain in synaptic efficacy [147,152,154,155]. This calibrated potentiation supports learning related plasticity while preserving network stability [152,153,154]. Preclinical models show restoration of LTP, improved memory, and elevated BDNF and synaptic proteins without evidence of neuronal injury or inflammatory activation [147,156]. Strengthening of inhibitory tone and increased seizure resistance further indicate a favorable excitation inhibition balance [153,155]. These properties make eEF2K a mechanistically interesting but still preclinical adjunct concept for fast-acting glutamatergic antidepressant research [147,154]. NMDA and AMPA modulators initiate rapid relief by triggering glutamate-driven plasticity, yet their benefits often fade as translational programs normalize [147,152,157]. By sustaining protein synthesis downstream, eEF2K inhibition could stabilize newly potentiated synapses and extend antidepressant duration [147,156,158]. Combining AMPA centered drive with translational disinhibition offers a rational strategy to deepen and prolong circuit remodeling while limiting overexcitation, particularly in TRD [49,147,152,155].

A major gap remains the lack of clinical grade-eEF2K inhibitors. Despite consistent neuroprotective- and plasticity-enhancing effects across preclinical disease models, all available compounds remain experimental, with unresolved issues in kinase selectivity, brain exposure, and chronic tolerability [159,160,161]. Advancing the field requires rigorous safety and target engagement strategies, including quantitative phosphorylated eEF2 readouts, pharmacokinetic–pharmacodynamics (PK-PD)-guided dosing, and early toxicity profiling across mitochondrial, cardiac, and genotoxic domains [159,160,162,163]. Equally important is avoiding global disruption of protein synthesis while restoring adaptive translation [161,162]. This limitation should temper translational interpretation. At present, eEF2K inhibition remains a preclinical strategy, and the field lacks clinical-grade compounds with proven brain penetration, kinase selectivity, and chronic safety. Because eEF2K sits within broader translational control networks, long-term modulation may carry risks related to off-target kinase activity, metabolic stress, seizure threshold changes, or maladaptive protein synthesis. Beyond chemistry, translational progress will depend on precision deployment [159,160,161]. eEF2K signaling intersects with excitation inhibition balance and circuit level plasticity, suggesting strong synergy with circuit specific interventions [159,160,164]. Combining eEF2K inhibition with neuromodulation, targeted NMDAR or AMPAR modulation, or timed cognitive training could stabilize translational homeostasis during defined plasticity windows [160,162]. Stratifying patients by circuit dysfunction and aligning drug exposure with network level engagement offers a rational path to clinical relevance and durable benefit [93,159,160,161]. This rationale remains provisional. No eEF2K inhibitor has yet established antidepressant efficacy in humans, and the absence of clinical-grade compounds limits assessment of target engagement, tolerability, and long-term safety [146,165]. Negative or null results should be expected as the field moves from pathway validation to drug development [165,166]. Accordingly, eEF2K should be discussed as a target for hypothesis testing, not as a near-term clinical strategy. This distinction is important because eEF2K inhibition remains a preclinical hypothesis, with no clinically viable inhibitor yet demonstrating antidepressant target engagement, safety, or efficacy in humans (Table 1).

## 3. Stabilizing Presynaptic and Network Integrity

Sustained antidepressant benefit requires presynaptic mechanisms that preserve release fidelity after postsynaptic remodeling. This section therefore focuses on vesicle cycling, active-zone function, metabolic support, and SV2A-linked presynaptic integrity as maintenance mechanisms rather than reintroducing the induction cascade [20,48,76,163,176,177,178,179,180,181,182,183]. Presynaptic stability is also influenced by non-neuronal and metabolic context. Astrocytes regulate glutamate clearance and energetic support, immune mediators reshape synaptic pruning and vesicle function, and systemic endocrine or inflammatory states may alter whether synaptic remodeling becomes adaptive or maladaptive.

### Synaptic Vesicle Glycoprotein 2A (SV2A) Enhancers

SV2A is a core presynaptic regulator that coordinates vesicle priming, calcium dependent release, and neurotransmitter loading to ensure reliable synaptic transmission [184,185,186]. Localized to synaptic vesicles, it aligns vesicle readiness with calcium triggered fusion, shaping the efficiency and timing of exocytosis without altering presynaptic calcium entry itself [186,187,188]. A defining feature of SV2A is its tight coupling to synaptotagmin, the principal calcium sensor for release [189,190,191]. By binding synaptotagmin and regulating its endocytic retrieval, SV2A ensures accurate recycling and vesicular packaging of this sensor, a requirement for synchronous release and high fidelity signaling [191,192,193]. SV2A also stabilizes vesicle content and maintains the readily releasable pool, thereby tuning short term plasticity and sustaining output during repeated activity [185,186,194]. Beyond single synapses, SV2A expression scales with synaptic density across cortical and limbic networks and serves as a proxy for presynaptic integrity [173,195,196]. Its influence is particularly pronounced at inhibitory terminals, where it shapes GABA release and constrains network hyperexcitability [173,187,197]. During periods of heightened plasticity, SV2A nanoclusters with synaptotagmin support rapid vesicle recycling and preserve presynaptic identity as demand for precise excitation secretion coupling increases [189,192,193]. Through these convergent actions, SV2A functions as a molecular scaffold that stabilizes presynaptic terminals while postsynaptic strengthening consolidates [175,184,198]. This places SV2A at the center of synaptic resilience, linking vesicle dynamics to durable circuit integrity in health and disease [173,175,195].

Across models of stress, epilepsy, and neurodegeneration, modulation of SV2A preserves synaptic structure and maintains transmission after plasticity induction [173,175,199]. Levetiracetam restores vesicle fusion fidelity, rescues basal transmission, and normalizes synaptic protein composition, effects that depend on direct SV2A engagement and extend to hippocampal volume and plasticity recovery [173,175,199]. PET ligands confirm that SV2A density indexes synaptic integrity; ketamine appears to elevate SV2A where baseline is low, aligning structural rescue with symptomatic improvement and suggesting a convergence between plasticity inducers and presynaptic stabilizers [173]. Brivaracetam, with higher affinity and rapid brain entry, similarly occupies SV2A in vivo, supporting a target occupancy to protection relationship that generalizes across compounds and species [200,201,202]. These actions are not purely neuronal [203,204]. Both agents reduce pathologic astroglial glutamate release, supporting glutamatergic homeostasis during periods of heightened network demand [203,204].

Functionally, SV2A modulation limits the slide from induced plasticity to maladaptive remodeling and relapse-like behavior [175,199,205]. In stress-sensitized and developmental perturbation paradigms, levetiracetam reverses anxiety-like, cognitive, and social deficits while rebalancing hippocampal and mesolimbic activity, consistent with network-level stabilization of prefrontal–hippocampal circuits [175,199]. After ketamine or other plasticity-promoting interventions, preserving vesicle cycling and maintaining a competent readily releasable pool appear crucial for sustaining gains; animal studies link antidepressant durability to synaptic integrity supported by SV2A pathways and complementary signaling through ERK and calcium channels [199,201,205]. Together, these data position SV2A as a presynaptic gatekeeper that consolidates structural and functional benefits after plasticity induction, mitigating synaptic loss and reducing behavioral recurrence across disease-relevant contexts [175,199,201].

PET targeting SV2A has matured into a practical readout of synaptic density in vivo, with carbon-11 (^11^C) and fluorine-18 (^18^F) ligands enabling cross-species translation [206,207,208]. [^11^C]UCB-J established high brain uptake and specificity, creating the benchmark for human and preclinical studies, while [^18^F]SynVesT-1 and [^18^F]SynVesT-2 extend accessibility through longer half-life, favorable kinetics, and validated simplified quantification protocols suited to clinical workflows [174,208,209]. In rodents and nonhuman primates, these tracers map regional SV2A with reliability and support longitudinal designs, including occupancy and therapeutic challenge paradigms [209].

Across disorders, SV2A PET consistently reveals synaptic loss [210,211,212]. Depression cohorts show lower binding with evidence that pharmacologic challenges can probe synaptogenesis in vivo [212]. In Alzheimer’s disease, widespread reductions, particularly in hippocampus and association cortex, correlate with cognitive impairment and track amyloid/tau/neurodegeneration (A/T/N) pathology; similar decreases emerge in Parkinsonian and other neurodegenerative conditions, underscoring presynaptic vulnerability across networks [206,211,213,214,215]. As a translational biomarker, SV2A PET is well-positioned to monitor antidepressant-induced synaptic restoration [215,216,217,218]. Proof-of-concept data demonstrate partial recovery after fluoxetine in a depression model, and early work suggests ketamine may normalize low-baseline SV2A signal, linking circuit plasticity to presynaptic stabilization [167,217,218]. Together, these advances support SV2A PET as an integrative tool for diagnosis, progression tracking, and therapeutic monitoring across neuropsychiatric disease [14,206,207,211]. However, SV2A PET has not yet been validated as a treatment-selection or dose-optimization tool for antidepressant care. Changes in tracer binding may reflect synaptic density, vesicle protein regulation, cellular composition, or methodological factors, and prospective studies are needed before SV2A PET can be used to guide clinical decisions. SV2A PET should be framed as a research biomarker for testing synaptic hypotheses, not as a clinically validated tool for antidepressant selection, dose optimization, or relapse prevention. Similar caution applies to EEG, fMRI, MRS, and peripheral biomarker panels, which remain useful for mechanistic studies but insufficient for routine patient stratification. Therefore, SV2A enhancers and SV2A PET should be framed as investigational maintenance-phase tools rather than validated antidepressant optimization strategies.

Levetiracetam analogs and newer SV2A enhancers are attractive maintenance-phase adjuncts because they directly stabilize presynaptic function after induction therapies [175,199,205]. Brivaracetam provides higher SV2A affinity, faster brain entry, and robust target occupancy, attributes that may consolidate network gains established by agents such as ketamine and reduce relapse risk through sustained vesicle cycling competence and dampening of pathological glutamate release [175,200,201,219]. Padsevonil and related ligands extend this pharmacology with optimized SV2A engagement and limited drug interaction profiles, positioning the class for chronic adjunctive use where synaptic resilience and clean tolerability are essential [175,219,220]. Preclinical and translational data also indicate that levetiracetam normalizes vesicle fusion and curbs amyloidogenic stress, with mitochondrial SV2A contributions to cognitive preservation, suggesting presynaptic and metabolic protection during maintenance [163,179,221,222,223].

Clinically, both levetiracetam and brivaracetam have favorable safety, simple kinetics, and flexible dosing that support long-term administration [224,225]. However, this clinical familiarity should not be equated with established antidepressant efficacy. SV2A ligands were developed primarily for epilepsy, and their repurposing for depression will require prospective trials that separate presynaptic stabilization from nonspecific sedation, cognitive effects, irritability, or mood lability [175]. Brivaracetam may show improved behavioral tolerability in some patients, and switching within the class is feasible when adverse effects emerge, enabling continuity of presynaptic stabilization [219,225]. PET studies verify dose-dependent SV2A occupancy at therapeutic levels, offering a pharmacodynamic bridge to personalized maintenance strategies and trial designs that couple synaptic target engagement with network connectivity metrics [200]. Conceptually, SV2A enhancers complement plasticity modulators by securing the presynaptic substrate needed to translate spine growth into durable circuit performance, a metaplasticity framework that aims to extend remission and minimize recurrence across neuropsychiatric disorders [205,226].

Progress will hinge on next-generation SV2A modulators that potentiate presynaptic function without distorting vesicle recruitment, priming, or endocytic sorting [189,192,227]. Current ligands confirm druggability, yet specificity remains blunt at the level of vesicle dynamics, raising concerns about activity-dependent depression, altered release probability, and unintended network dampening during chronic use [189,192,227]. Medicinal chemistry should prioritize bias for physiological coupling with synaptotagmin pathways, sparing mechanisms that constrain the readily releasable pool [192,228,229]. Parallel pharmacology needs rigorous off-target screens and in vivo assays that capture high-frequency transmission and metabolic resilience across cortical and limbic circuits [192,230,231].

Key design questions are unresolved. What dosing windows best stabilize presynaptic fidelity after induction therapies, and do these windows shift with age, hormonal state, or comorbidity? [232,233,234]. Sex differences in pharmacokinetics and plasticity trajectories remain largely unmapped, despite evidence that network biomarkers and treatment prediction are sensitive to demographic and biological heterogeneity [93,232,233,234]. Longitudinal SV2A PET is poised to answer these gaps, but validation demands harmonized kinetic modeling, test–retest data across centers, and standardized reference strategies from mouse to human cohorts [206,214,235,236]. The field should converge on multimodal trials that combine SV2A-targeted maintenance with plasticity inducers, while tracking synaptic density, dynamic connectivity, and behavioral endpoints to build durable, relapse-resistant network resilience [206,231].

## 4. Intrinsic Excitability Tuning (Gain Control)

**Figure 2:**
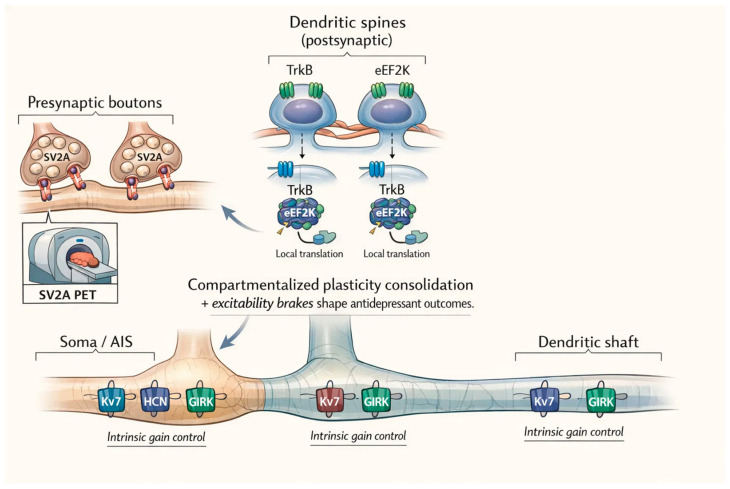
Synapse-to-spike targets for plasticity consolidation and gain control. This schematic maps key antidepressant-relevant targets onto a cortical pyramidal neuron. At dendritic spines, TrkB supports consolidation by linking transient potentiation to stable spine maturation, while eEF2K acts as a local translation checkpoint that regulates protein synthesis needed for synaptic persistence. At presynaptic boutons, SV2A stabilizes vesicle cycling and release fidelity, providing a bridge between synaptogenic induction and maintenance of neurotransmission. Across the soma and dendrites, Kv7, HCN, and GIRK channels regulate intrinsic excitability by tuning firing threshold, dendritic integration, resonance, and inhibitory tone. Together, these targets illustrate how antidepressant discovery can coordinate synaptic remodeling with circuit-level gain control. eEF2K, eukaryotic elongation factor 2 kinase; GIRK, G protein-gated inwardly rectifying potassium channel; HCN, hyperpolarization-activated cyclic nucleotide-gated channel; Kv7, voltage-gated potassium channel subfamily Q; SV2A, synaptic vesicle glycoprotein 2A; TrkB, tropomyosin receptor kinase B.

Intrinsic excitability refers to the non-synaptic control of how neurons translate inputs into spikes, operating alongside synaptic plasticity to set firing probability, temporal precision, and network gain [237,238,239]. These channels shape how neurons convert input into firing across prefrontal, hippocampal, and mesolimbic circuits [237,238,240]. In this context, three modulatory systems emerge as central governors of neuronal tone and promising antidepressant targets. Kv7 channels provide a non-inactivating outward current that restrains repetitive firing and supports gamma-rhythmic coordination; pharmacological inhibition or enhancement shifts excitability and cognitive performance with clear translational leverage [238,241,242]. HCN channels, through hyperpolarization-activated current (Ih), set resting membrane potential and dendritic integration; their trafficking and microdomain targeting regulate affective behavior, with selective modulation yielding antidepressant-like effects while sparing cardiac liabilities [237,239,242]. GIRK channels dampen excitability downstream of G protein signaling (RGS); their partial inhibition elevates firing and produces antidepressant-like actions, highlighting a therapeutically tractable brake on network activity [243,244,245]. The section proceeds from mechanistic principles to translation. I first outline how Kv7, HCN, and GIRK currents gate excitability and co-regulate with synaptic scaling. This section then synthesizes preclinical evidence across stress and antidepressant models, before surveying emerging clinical probes and candidate modulators. Finally, dosing and safety considerations are mapped to inform integrative strategies for durable circuit resilience. To maintain symmetry with the synaptic plasticity sections, HCN and GIRK channels are discussed not only as electrophysiological regulators, but also as circuit-level targets with distinct regional logic, translational opportunities, and safety constraints. Throughout this section, a distinction is made between established neurophysiological roles of Kv7, HCN, and GIRK channels, antidepressant-like effects in preclinical models, and clinically validated antidepressant mechanisms. At present, the strongest clinical signal is limited to early Kv7 work, whereas HCN and GIRK strategies remain largely mechanistic or preclinical. Thus, intrinsic excitability modulation is proposed mainly as an adjunctive maintenance and relapse-prevention strategy, not as a stand-alone primary antidepressant mechanism. This distinction also separates intrinsic excitability from synaptic plasticity: plasticity changes synaptic strength or structure, whereas intrinsic excitability changes how neurons convert input into firing output. In the ICM framework, synaptic plasticity explains how connections are modified, whereas intrinsic excitability explains how remodeled neurons convert input into firing output. Their interaction is therefore complementary: plasticity rewires circuit architecture, while excitability tunes the gain and stability of the remodeled circuit. Figure 2 is designed to localize candidate targets along the synapse-to-spike axis, but it should not be interpreted as evidence that all depicted mechanisms have equal clinical validation.

### 4.1. Voltage-Gated Potassium Channel Subfamily Q (Kv7) Openers

Kv7 channels formed by potassium voltage-gated channel subfamily Q member 2–5 (KCNQ2–5) subunits generate the M-current (muscarinic-sensitive potassium current), a non-inactivating conductance that opens near resting membrane potential, stabilizes membrane potential, and raises the threshold for spike initiation [246,247,248]. By providing a steady outward conductance at the axon initial segment, these channels curb afterdepolarizations and limit high-frequency firing; loss of function or reduced membrane targeting disrupts this brake and promotes pathological bursting with cognitive and seizure phenotypes [242,248,249]. Molecular studies show that phosphatidylinositol-4,5-bisphosphate and small-molecule openers such as retigabine bias the pore toward the open state, offering a structural rationale for pharmacologic control of excitability [246]. Conversely, closure via Gq-coupled receptor pathways or direct blockers like paroxetine and linopirdine depolarizes neurons and increases release probability, a double-edged mechanism that can enhance cognition yet risk hyperexcitability when unchecked [245,246,247].

Within prefrontal and limbic circuits, Kv7 activity normalizes neuronal gain and suppresses stress-evoked firing escalation that impairs working memory and mood regulation [241,250,251]. In rodent cortex and hippocampus, openers reduce burst propensity, rescue hyperexcitability and seizure-related mortality, and limit excitotoxic injury after traumatic brain insult, underscoring disease-modifying potential [242]. Together, these data position Kv7 channels as nodal determinants of intrinsic excitability whose activation stabilizes network dynamics and protects mood-relevant circuits from maladaptive bursting [248,249,251].

Kv7 openers consistently dampen hyperexcitability, promote stress resilience, and stabilize affective behavior across rodent paradigms. Retigabine and related ligands shift channel activation toward more negative potentials, increase resting conductance, and suppress burst firing, which translates into robust antidepressant-like and mood-stabilizing effects after chronic social defeat and other stressors [245,252,253]. In the ventral hippocampus and ventral tegmental area (VTA), pharmacologic activation or genetic upregulation of potassium voltage-gated channel subfamily Q member 2 (Kcnq2) normalizes pathological firing and restores social interaction and anhedonia metrics; adjunct retigabine amplifies ketamine’s sustained benefits, linking intrinsic excitability control to durable antidepressant action [245,253,254]. Pan-selective Kv7 openers such as Lu AA41178, a brain-penetrant Kv7.2–Kv7.5 potassium channel activator, extend these findings, reducing depressive-like behavior while elevating seizure thresholds without major off-target liabilities, suggesting a favorable translatability profile for network stabilization [255,256].

Prevention of relapse aligns with neuroprotection. The Kv7 potassium channel enhancer QO-83, a small-molecule KCNQ/Kv7 channel opener, limits infarct volume, edema, and cognitive decline after ischemic injury while curbing microglial activation, pointing to attenuation of excitotoxic cascades that often follow stress or drug withdrawal [252,255,256,257]. Notably, agents that inhibit Kv7, including paroxetine at relevant concentrations, increase excitability and could heighten vulnerability to rebound hyperexcitability when plasticity inducers are tapered, underscoring the mechanistic rationale for Kv7-guided maintenance strategies [247,248,258]. Converging data across serotonergic, hippocampal, and mesolimbic nodes further indicate anxiolytic actions of Kv7 activation, consolidating a preclinical case for mood stabilization and relapse prevention through targeted M-current augmentation [245,247,253].

Human data with Kv7 openers provide a limited but informative clinical signal, rather than definitive validation of Kv7 modulation as an antidepressant strategy. In major depressive disorder with prominent anhedonia, ezogabine produced meaningful reductions in depressive symptoms and improved reward sensitivity in randomized and open-label studies, with parallel changes in ventral striatal connectivity and reward learning [259,260,261]. These observations align with preclinical evidence that Kv7 activation restores resilience in stress-sensitized networks and dampens pathological hyperexcitability, suggesting particular relevance for patients with affective lability, dysphoric agitation, or anxiety-driven reward blunting [253,261,262]. Beyond mood, ezogabine reduces cortical and spinal motor neuron excitability in amyotrophic lateral sclerosis and decreases seizure frequency in refractory epilepsy, demonstrating target engagement in human hyperexcitable states and supporting its transdiagnostic potential to quiet unstable circuits [256,262,263].

Clinical translation is not without friction. Chronic ezogabine is associated with dose dependent dizziness, somnolence, confusion, urinary retention, and, most notably, blue or purple pigmentation of retina, nails, and skin due to drug and metabolite accumulation in melanin rich tissues [253,258,262]. These liabilities, along with the need for urologic and ophthalmologic monitoring, ultimately limited widespread use. Still, the pharmacology remains compelling. Retigabine and newer derivatives can attenuate negative affect, reduce anxiety like states, and suppress maladaptive reward seeking, including cocaine self-administration, while supporting longer term stabilization of excitability [254,261,264]. These data support further testing of Kv7 openers in selected relapse-prone or affectively labile subgroups, but they do not yet establish this class as a broad antidepressant strategy [254,259,261]. A further limitation is that the most clinically informative Kv7 opener, ezogabine or retigabine, was constrained by tolerability and tissue pigmentation concerns, which limits direct psychiatric translation. Newer Kv7.2/7.3-biased compounds may overcome some liabilities, but antidepressant efficacy, long-term safety, and optimal phenotype selection remain unresolved.

Next-generation Kv7 modulators are converging on greater selectivity, cleaner safety, and circuit-aware deployment. Structure-guided chemistry and in silico design have produced Kv7.2/7.3-biased agonists with order-of-magnitude potency gains over retigabine, improved pharmacokinetics, and reduced liabilities linked to earlier chemotypes [265,266,267]. Brain-penetrant exemplars such as Lu AA41178 and SCR2682, pan-Kv7 (KCNQ2–5) potassium-channel activators, demonstrate broad antiexcitability efficacy without major off-target activity, while clinical candidates including azetukalner and BHV-7000, Kv7.2/7.3-selective channel activators, aim to retain efficacy while avoiding pigmentation and urinary liabilities associated with earlier chemotypes [242,262,267]. Subtype control is now tractable: minimal substitutions can invert activity across isoforms, enabling selective activation of Kv7.2/7.3 over Kv7.4/7.5 and allowing circuit-tailored effects [242,266,268]. Natural-product leads like echinocystic acid and endogenous modulators such as dehydroepiandrosterone sulfate (DHEAS) expand chemotype space and suggest allosteric stabilization strategies that preserve physiological gating [267,269].

Therapeutically, combinatorial approaches should pair Kv7 openers with plasticity inducers to convert rapid symptom relief into durable remission. Targeted Kv7 activation can stabilize VTA–nucleus accumbens (NAc) and prefrontal ensembles after induction, limit rebound hyperexcitability and extend benefit windows; dual-target constructs that couple Kv7.2/7.3 agonism with transient receptor potential vanilloid 1 (TRPV1) inhibition offer further resilience without added side effects [242,254,270]. Rational sequencing with synaptogenic agents and circuit-specific delivery will be central to translation [262,265,267].

### 4.2. Hyperpolarization-Activated Cyclic Nucleotide-Gated (HCN) Channel Modulators

HCN channels open with membrane hyperpolarization to conduct the mixed cation current Ih, a slowly activating inward flux that depolarizes dendrites, lowers input resistance, and tightens the temporal window for integration [271,272]. Enrichment of HCN channels in distal apical tufts equips pyramidal neurons with band-pass properties, so inputs near theta and low beta frequencies are preferentially transmitted while slower components are shunted [273,274,275]. This resonance is not hard-wired [271,272,276]. Ih is tuned by cAMP and the auxiliary subunit tetratricopeptide repeat–containing Rab8b-interacting protein (TRIP8b), and it cooperates with inwardly rectifying potassium channel subfamily 2 (Kir2) and M-type K^+^ conductances to set effective gain [276,277,278]. At the network level, hyperpolarization-activated cyclic nucleotide-gated channel 4 (HCN4) sustains thalamocortical rhythms that scaffold cortical timing, linking molecular gating to mesoscale oscillations [272,277,279]. Depending on dendritic location and partner channels, Ih can either dampen or sharpen excitatory postsynaptic potentials (EPSPs), yielding a context-dependent balance of excitatory and inhibitory effects that stabilizes activity yet preserves rapid responsiveness [273,274,280]. Together, these mechanisms establish oscillatory gain control from single dendrites to distributed circuits [271,272]. A deeper translational distinction is that HCN modulation is not simply pro-excitatory or anti-excitatory. Its behavioral effect depends on isoform composition, dendritic compartment, and circuit node. In distal dendrites, HCN1-rich conductance can constrain temporal summation and tune resonance; in thalamocortical and hippocampal loops, HCN-dependent timing can shape oscillatory coupling, cognitive flexibility, and internally oriented network states. This makes HCN channels promising but difficult targets: partial, region-aware modulation may normalize rumination-like or cognitive-inertia phenotypes, whereas indiscriminate blockade may impair working memory, anxiety regulation, or cardiac rhythm.

In prefrontal cortex, hyperpolarization-activated cyclic nucleotide-gated channel 1 (HCN1)-driven Ih stabilizes intrinsic persistent firing and supports working memory by maintaining depolarized up-states and filtering distractors within resonant frequency bands [275,277,281]. Developmental increases of Ih in pyramidal neurons, along with cell-type specific regulation by synaptic plasticity and fragile X mental retardation protein (FMRP), refine this gain control and may delineate windows of vulnerability to cognitive dysfunction [277,278,281]. Computational and experimental studies in layer V show that Ih facilitates proximal inputs while constraining distal summation, thereby shaping how rhythmic afferents from limbic and thalamic sources influence cortical excitability [273,274,280]. Through this bidirectional, location-sensitive control, Ih tunes the impact of oscillatory drive on mnemonic maintenance and modulates emotional tone by gating limbic–prefrontal coupling [275,281]. Dysregulated HCN signaling alters resonance and timing, contributing to network states linked to mood and executive symptoms, whereas targeted modulation promises restoration of frequency-specific gain without sacrificing the dynamical flexibility essential for adaptive cognition [272,277].

Across preclinical systems, convergent evidence indicates that tempering Ih via partial HCN downregulation can normalize large-scale dynamics associated with perseverative self-focus [282,283,284]. Causal manipulations that attenuate default-mode drive reduce its pathological coupling: optogenetic silencing of the lateral habenula diminishes default mode network (DMN) hyperconnectivity in a depression model, while chemogenetic suppression of anterior cingulate cortex reconfigures DMN edges and improves behavior aligned with reduced rumination liability [284,285,286]. Molecular levers point the same way [167,283,287]. In post-stroke mice, hippocampal HCN1 inhibition lowers Ih, suppresses NOD-, LRR-, and pyrin domain–containing protein 3 (NLRP3) signaling, and ameliorates depression- and anxiety-like phenotypes; chronic hippocampal cAMP elevation similarly reduces HCN surface expression and rescues stress-induced cognitive deficits, consistent with a network shift away from internally oriented attractor states [282,283,287]. A brain-penetrant HCN blocker produces antidepressant-like effects, supporting target validity while underscoring the need for dose-limited, partial modulation [283,288].

Region and circuit specificity remain pivotal [283,288,289]. HCN1 upregulation in lateral habenula drives anxiety during morphine withdrawal, and its inhibition or knockdown reduces this burden [289]. By contrast, antagonizing HCN channels in ventral tegmental dopamine neurons or blocking HCN in basolateral amygdala prolongs inhibition and heightens anxiety, cautioning against indiscriminate blockade [283,288]. Network-level corroboration comes from white-matter disruption of forceps minor that perturbs DMN connectivity, increases anxiety, and normalizes with recovery, linking DMN integrity to affective behavior and strengthening the translational rationale for calibrated HCN modulation to curb rumination-like and anxiety-related outcomes [284,285].

Available evidence suggests a plausible HCN-linked phenotype involving cognitive inertia and slowed affective transitions, but this remains a translational hypothesis rather than a validated treatment mechanism [290,291]. Clinically, dorsolateral prefrontal transcranial direct current stimulation (tDCS) yields the largest benefits in patients with psychomotor retardation and executive disturbance, and meta-analytic datasets suggest comparable efficacy to several standard treatments when dose and resistance are considered, with emerging hints of cognitive improvement alongside mood change [290]. These responders likely benefit from rebalancing large-scale networks in which Ih tunes prefrontal resonance and thalamo-hippocampal drive [291,292]. Converging preclinical work strengthens the mechanistic bridge: chronic stress elevates HCN1 and Ih in dorsal Cornu Ammonis area 1 (CA1), reducing excitability and producing depressive behaviors, while lowering HCN surface expression via cAMP signaling or direct HCN1 inhibition rescues stress-related cognitive deficits and post-stroke affective slowing [289,292,293]. Medicinal chemistry efforts now pursue brain-biased ligands and TRIP8b-guided strategies to avoid cardiac liabilities, and first-generation brain-penetrant HCN inhibitors reverse social and cognitive susceptibility in mice [283,294,295]. In parallel, circuit-level interventions such as tDCS, and potentially deep brain stimulation (DBS) targeting medial prefrontal–subgenual loops, offer complementary leverage over HCN-sensitive pathways [290,291].

Current pharmacology still lacks truly isoform-selective HCN modulators, which constrains both inference and dosing [296,297]. Canonical blockers such as ivabradine, a clinically approved HCN channel inhibitor, and ZD7288, a widely used experimental HCN channel blocker, bind within the HCN1 pore with modest affinity and broad cross-reactivity, while many “isoform-preferring” tools have incomplete pharmacokinetic and off-target profiling, resulting in narrow therapeutic windows and variable central effects [277,298]. Structural and biophysical advances point to a path forward. High-resolution conductance measurements across homomeric and heteromeric channels, coupled with emerging cyclic nucleotide–binding domain (CNBD)-focused screening platforms and auxiliary-subunit aware design, provide blueprints for subtype and state selectivity that remain to be realized in vivo [299,300]. Until such agents mature, trials need mechanistic readouts that scale from rodents to humans [291]. Electroencephalography (EEG) offers direct indices of Ih-linked resonance, including theta coherence, right frontal theta during control, and slow-wave power during sleep; seizure-model signatures and threshold-tracking paradigms add sensitivity to network instability [291]. Complementary functional magnetic resonance imaging (fMRI) metrics, particularly resting-state DMN coupling and task-evoked deactivation, can register circuit-level normalization. Integrating these EEG–fMRI biomarkers into early-phase studies will de-risk development and anchor dose selection to HCN biology [291]. This limitation also explains why HCN strategies remain less clinically mature than ketamine-linked plasticity approaches. Future work should define whether HCN modulation is best deployed as a pharmacological intervention, a neuromodulation-sensitive circuit readout, or a biomarker-guided adjunct for patients with cognitive slowing, rumination, or altered theta-band dynamics. Accordingly, HCN modulation should be framed as a candidate circuit mechanism that requires isoform-selective tools, brain-region specificity, and prospective clinical testing before antidepressant relevance can be inferred.

### 4.3. G Protein-Gated Inwardly Rectifying Potassium Gated Channel (GIRK) Openers

GIRK channels are G protein-gated inwardly rectifying potassium conductances that convert inhibitory GPCR signaling into membrane hyperpolarization and reduced input resistance [301,302,303]. At pyramidal cell dendrites and somata, activation by gamma-aminobutyric acid type B (GABA_B) and 5-hydroxytryptamine receptor 1A (5-HT_1_A) receptors releases G protein βγ (Gβγ) to open inwardly rectifying potassium channel subfamily 3 (Kir3.x) pores, lowering excitability and truncating EPSP duration, while preserving temporal precision of synaptic drive [301,302,303]. The resulting outward K^+^ flux restores baseline tone during sustained neuromodulatory input and prevents runaway firing during high synaptic load [302,304,305]. Mechanistically, signaling gain and kinetics are sculpted by regulator of RGS proteins of the R7 family, which accelerate guanosine triphosphatase (GTPase) activity and impose receptor selectivity, by co-activation of convergent inhibitory GPCRs that sum on GIRK, and by intracellular Na^+^ that boosts GIRK2 gating during bursts [301,306,307]. Channel activity is further tuned by redox state and direct lipid or small molecule interactions [302,306] Pharmacology underscores tractability: a GIRK1-selective opener and other direct activators hyperpolarize hippocampal pyramidal neurons and reduce seizure susceptibility, and photoswitchable openers provide millisecond control of inhibitory tone for circuit dissection and therapeutic prototyping [301,305,308].

By reinstating a K^+^-dependent safety brake, GIRK channels rebalance hyperactive pyramidal ensembles and dampen circuit noise that degrades computation [304,305]. Increasing GIRK conductance lowers variance of subthreshold fluctuations, suppresses spontaneous bursting, and raises the signal-to-noise ratio for behaviorally relevant inputs. In hippocampal and cortical circuits, GIRK-dependent inhibition supports forms of plasticity that require stable excitability set points, and selective perturbation of GIRK activity in CA1 pyramidal neurons disrupts learning, highlighting its role in cognitive control [304,305,309]. When inhibitory reserve is compromised, as in early amyloid-β pathology or disease states with GIRK2 mislocalization, pyramidal hyperexcitability and oscillatory instability emerge; restoring GIRK function counters these phenotypes and normalizes inhibitory long-term plasticity [310,311,312]. Emerging therapeutics that enhance GIRK opening therefore offer a principled route to quiet pathological activity without silencing computation [301,305,313]. In concert with endogenous GABA_B and 5-HT_1_A signaling, and aided by domain-targeted modulators, GIRK channels provide a tunable lever for homeostatic gain control across limbic and associative networks [302,304]. GIRK channels also merit fuller consideration because they provide a receptor-coupled route for stabilizing excitability rather than directly forcing synaptic remodeling. Their position downstream of GABA_B, 5-HT1A, opioid, and other inhibitory GPCRs allows them to integrate neuromodulatory context with potassium conductance. In depression-relevant circuits, this may be especially relevant to agitation, threat reactivity, insomnia, and stress-sensitized noise, where the therapeutic aim is not to silence neurons but to restore inhibitory reserve and improve signal fidelity.

Pharmacological activation of GIRK channels delivers a coherent anti-agitation profile by lowering pyramidal cell gain and stabilizing limbic rhythms [305,314]. Selective GIRK1/2 channel openers, including ML297, a prototype small-molecule GIRK1/2 activator, and GAT1508, a next-generation brain-penetrant GIRK1/2 modulator, hyperpolarize principal neurons, reduce avoidance and anxiety-like behaviors, and facilitate extinction of conditioned fear without motor or cardiac liabilities, consistent with strengthened stress resilience and cleaner signal transmission through amygdalo-hippocampal pathways [314,315]. Direct GIRK agonists that bypass receptors, including GIRK agonist 1 (GiGA1), a direct GIRK channel activator, suppress seizure severity and agitation by curbing network hyperexcitability, while chemogenetic evidence confirms that GIRK conductances mediate GPCR-driven reductions in excitability in striatal circuits [316,317]. In prefrontal cortex, stress weakens GIRK1 signaling and impairs cognitive flexibility; restoring GIRK tone with ML297 rescues performance, positioning GIRK activation as a mechanistic counterweight to stress-sensitized noise in associative networks [318,319].

Sleep stabilization emerges as a complementary benefit. ML297 increases non–rapid eye movement (NREM) time, reduces wakefulness, and mimics GABA_B-linked sleep regulation, while brain-biased GIRK modulators similarly promote NREM in rodent models [305,320]. At the pacemaker level, GIRK2 is required for melatonin’s suppression of suprachiasmatic activity and for circadian phase shifts, anchoring a causal link between GIRK opening, decreased arousal, and normalized sleep–wake architecture [321]. Balanced activation remains essential, as excessive GIRK drive can perturb plasticity; nevertheless, calibrated GIRK enhancement reliably dampens limbic excitability and supports restorative sleep states in preclinical systems [304,322].

By converting inhibitory GPCR signals into stabilizing potassium currents, GIRK modulators can quiet hyperactive cortical–limbic ensembles while preserving information throughput, an effect profile that differs from global central nervous system (CNS) suppressants [305,323]. Early small-molecule openers illustrate this promise in preclinical anxiety and seizure models, suggesting that region- and subunit-selective targeting could attenuate arousal and irritability without cognitive blunting or abuse liability, a key consideration for agitated depression and mixed presentations [315,323]. Clinical sleep interventions can reduce insomnia and may improve mood or suicidal ideation, but these findings should not be taken as direct evidence for GIRK-targeted antidepressant efficacy [324,325,326]. Pharmacologic hypnotics can reduce ideation in severe insomnia when carefully deployed, yet their complex risk profile argues for circuit-tuned alternatives [324,325]. Brief behavioral insomnia therapies likewise improve sleep and mood in suicidal patients, supporting a stepped approach in which GIRK-guided, sedative-yet-non-suppressive modulation complements sleep-focused interventions to stabilize affect and lower acute risk [324,326,327].

Progress is constrained by a thin toolbox. Most GIRK activators show modest subtype resolution, incomplete coverage of heterotetramers, and uneven brain exposure, which complicates dose selection and inflates off-target risk [301]. Even exemplars illustrate the gap: ML297 favors GIRK1-containing channels but only partially discriminates subunit context and exhibits suboptimal CNS penetration; newer scaffolds such as GAT1508, VU0810464, a neuronal-biased GIRK channel opener, and VU0529331, a next-generation GIRK activator, improve neuronal bias yet still face selectivity and pharmacokinetic liabilities [301]. Structure-guided discovery is beginning to change the landscape. Cryo-EM views of PIP_2_ regulation, G protein family interfaces, and femtomolar-scale conductance features now inform SAR and virtual screening, enabling identification of GIRK1-preferring activators like GiGA1 and rare isoform-specific tools. Nevertheless, BBB constraints remain a central translational hurdle [126].

A practical pipeline should braid high-resolution modeling with medicinal chemistry for allosteric pockets that encode state and subunit selectivity, then validate across heteromeric compositions and red-flag panels [316]. Parallel work must incorporate brain-relevant permeability screens and predictive BBB models early, rather than late rescue. Circuit precision will also matter: chemogenetic or ligand-directed delivery can focus GIRK enhancement to limbic and prefrontal ensembles that drive agitation and sleep disruption while sparing brainstem and cardiac populations [301]. Finally, assay batteries should pair ion-channel pharmacology with translational readouts, including EEG markers of network stability and fMRI connectivity metrics, to anchor mechanistic engagement before large trials [301]. Compared with Kv7 channels, GIRK channels remain at an earlier translational stage. The main obstacles are subtype selectivity, heterotetramer complexity, brain exposure, and the need to avoid excessive inhibitory tone that could impair learning or motivation. A more symmetrical development path would therefore pair GIRK pharmacology with EEG sleep architecture, arousal metrics, anxiety and agitation phenotyping, and fMRI measures of limbic-prefrontal stability (Table 2).

## 5. Multi-Point Strategies and Combinatorial Approaches

Enduring antidepressant effects rarely arise from a single molecular nudge; they emerge when plasticity is first opened, then consolidated, and finally stabilized across interconnected networks. Depression reflects distributed circuit dysfunction that spans hippocampus, prefrontal cortex, and thalamocortical loops, so a credible pipeline must act at multiple nodes rather than chase a lone receptor. Rapid-acting agents and neuromodulation converge on metaplasticity, the regulation of how readily plasticity can later be induced, and BDNF–TrkB signaling, creating a time window of enhanced rewiring capacity that behavioral inputs can shape toward healthier attractor states [332,333]. Within this framework, three tiers organize translation. First, plasticity plus stabilizers: pair inducers of iPlasticity, a juvenile-like reopening of experience-dependent plasticity, or TrkB activation with homeostatic mechanisms that prevent rebound noise and consolidate new connectivity, for example lithium, activity-dependent routines, or interneuron-targeted TrkB engagement [334]. Second, Plasticity plus excitability control: combine agents that restore synaptic strength with conductance-level brakes that normalize gain during reconnection, such as selective channel modulators to temper hyperexcitability while plasticity unfolds [333,335]. Third, AMPA-facilitating add-ons: bolster glutamatergic throughput and synapse stabilization by enhancing AMPAR trafficking or receptor drive, as illustrated by tianeptine, which promotes AMPAR trafficking and mTOR-linked synaptic plasticity, and by mGlu5-to-AMPAR coupling mechanisms that amplify downstream plasticity signaling [336,337]. The rationale is integrative. Pharmacology sets the stage, targeted neuromodulation aligns oscillatory gateways, and structured behavioral experiences write the final pattern, turning short-lived plasticity into durable network recovery [332,335].

### 5.1. Plasticity Core + Stabilizers

Rapid antidepressant action can be conceptualized as a staged sequence: glutamate-driven induction, TrkB- and translation-dependent consolidation, and presynaptic or excitability-based maintenance [54,338]. The detailed molecular cascade has been presented earlier; here the emphasis is on therapeutic sequencing. Durable benefit, however, requires a consolidation phase that secures these nascent changes. TrkB activation triggers ERK and mTORC1 cascades, extends protein synthesis windows, and supports dendritic spine maturation; methyl-CpG-binding protein 2 (MeCP2)-linked transcriptional programs lock in circuit reconfiguration over days [56,339]. In parallel, stabilization of presynaptic architecture indexed by SV2A helps restore synaptic density in patients with low baseline SV2A, aligning molecular repair with symptom relief [47,340]. Reinforcing these early gains prevents networks from sliding back into high-inertia, internally focused attractor states characteristic of depression. Practical implementations layer induction with targeted consolidation: ketamine or esketamine to open plasticity, followed by strategies that maintain TrkB signaling, sustain eEF2K inhibition within physiological bounds, and preserve synaptic vesicle competence via SV2A. Such sequencing links rapid glutamatergic rebalancing to structural and transcriptional maintenance, converting short-lived potentiation into persistent normalization of connectivity and affective dynamics [51,97].

Across animal models, pairing a fast plasticity inducer with a consolidation enhancer consistently converts transient synaptic gains into durable remission. Induction can be achieved with ketamine or AMPA-potentiating ampakines of the CX series, which rapidly boost Ca^2+^-permeable AMPAR drive, elevate BDNF, and engage mTOR-ERK pathways while sparing Hebbian learning capacity [341,342]. Consolidation then extends and stabilizes these changes: TrkB agonism or positive modulation with 7,8-DHF restores thin spine maturity, prevents stress- or age-related synaptic erosion, and sustains cognitive recovery across hippocampal, amygdalar, and cortical circuits [343,344]. Direct combinations are especially informative. In chronic stress paradigms, (R)-ketamine co-administered with LY341495, a selective group II mGlu2/3 antagonist, yields rapid and long-lasting behavioral improvement through BDNF-TrkB and AMPAR mechanisms, with synaptogenesis that outlasts single-agent effects [342,345]. Ketamine or its metabolite 2R,6R-HNK induces enduring AMPAR remodeling in mesolimbic pathways, while boosting ERK activity further prolongs antidepressant responses for weeks to months, indicating that downstream reinforcement is both necessary and sufficient for durability [341,346]. Postoperative and anesthesia-related depression models show similar synergy, where ketamine plus TrkB-dependent signaling reverses synaptic loss and maintains affective recovery [344].

Head-to-head work underscores the principle: agents that combine robust induction with TrkB-linked consolidation produce larger, longer-lived gains in spine density, fewer relapses after stress re-exposure, and superior functional rescue compared with either strategy alone [343].

Clinical translation benefits when pharmacological induction of plasticity is precisely coupled to consolidation via sleep, circadian alignment, psychotherapy, and neuromodulation. Rapid antidepressant responses need an induction phase followed by sleep-driven stabilization, orchestrated by slow oscillations, spindles, and ripples that support replay and synaptic renormalization [243,332]. Multi-component sleep and circadian programs improve psychiatric outcomes when practiced at biologically suitable times, with phase-sleep coupling mediating symptom change and durability from youth cohorts to depressive disorders [243,347]. Targeting circadian timing with light, activity schedules, melatonin, and dose timing can open or extend windows of plasticity; digital phenotyping personalizes scheduling, while mixed results from intensive care unit (ICU) circadian-intervention studies emphasize the importance of protocol fidelity to circadian biology [348,349]. Psychotherapy should be layered onto these windows. Cognitive enhancers and memory modulators administered just before or shortly after exposure-based work can gate encoding and reconsolidation, though agent choice and minute-scale timing remain decisive, particularly in trauma-focused care with mixed efficacy [350,351]. Sequential integration of pharmacotherapy followed by structured cognitive behavioral therapy (CBT) reduces relapse, likely converting state-dependent gains into enduring skills [351]. Repetitive transcranial magnetic stimulation (rTMS) or intermittent theta-burst stimulation (iTBS), delivered alone or in combination with medications, increases cortical plasticity and strengthens therapeutic effects when paired with task engagement, especially in individuals with higher baseline plasticity [351,352].

Translational pairings can extend rapid symptom gains into durable remission. Ketamine produces swift mood elevation, while iTBS supplies a modifiable scaffold whose effects often persist longer than infusion benefits, inviting sequencing to prolong response and minimize relapse [353,354]. Accelerated iTBS, delivered in clustered daily sessions, is safe, fast acting, and well suited to the first days after ketamine when metaplastic windows are most permissive [353]. Real-world multisite data confirm effectiveness and safety of left prefrontal iTBS, supporting routine integration into combinatorial care [353,354]. A practical map would initiate ketamine to unlock synaptic potentiation, then deliver targeted iTBS with symptom-contingent tapering and session timing aligned to circadian stability, aiming to convert state change into trait resilience [353].

Pharmacologic pairing follows the same logic. DXM with bupropion brings NMDA modulation, sigma-1 signaling, and monoaminergic support, offering a versatile backbone for augmentation [355,356]. Combination therapy outperforms monotherapy in many contexts, yet bupropion pairings show heterogeneous effects, which argues for precision add-ons and patient stratification [356]. Inhibiting eEF2K could amplify translation and synaptic strengthening downstream of glutamatergic modulation, creating a plausible synergy with DXM-bupropion that merits phase Ib signal-seeking trials [355,356]. Multimodal designs should incorporate pharmacokinetics, target engagement biomarkers, adaptive randomization, and personalized scheduling of behavioral activation and sleep regularization around predicted peaks of plasticity for each participant.

### 5.2. Plasticity + Excitability Control

Plasticity induction is not purely beneficial; without constraint it can push networks toward hypersynchrony, unstable bursting, and loss of information fidelity. Homeostatic mechanisms counter this drift by sensing activity history and restoring gain around an operating point through synaptic scaling and intrinsic adjustments [357,358]. After deprivation or strong potentiation, neurons upregulate hyperpolarization-activated currents and reshape burst dynamics, a response that curbs run-away excitation and normalizes firing statistics [357,359]. Excess extracellular glutamate shifts in inhibition, or impaired chloride handling can otherwise widen excitability, erode signal-to-noise, and degrade learning rules [360,361]. At the network level, connectivity is rebalanced to preserve stable population activity, yet this compensation is slow and incomplete without intrinsic brakes [358].

Ion channel tuning provides that brake and preserves gain precision. Kv7 channels furnish a non-inactivating M-current that stabilizes the axon initial segment; redistribution or pharmacologic enhancement reduces spurious spiking and limits burst afterdepolarizations [360,362]. Conversely, agents that inhibit Kv7 can transiently widen plasticity but risk hyperexcitability unless paired with compensatory controls [360,363]. HCN channels add negative feedback through Ih, accelerating membrane recovery and dampening resonance that seeds pathological bursting [357,359]. GIRK channels hyperpolarize the membrane and raise rheobase, offering a tractable target for post-induction stabilization [361]. Additional levers refine this clamp: GABAergic axo-axonic input tunes initial segment structure and thresholds, SK2 gating sculpts spike clusters, endocannabinoid-driven Kv7 augmentation quiets circuits after LTD, and ERK-linked control of Kv7.3 aligns molecular state with excitability demands [358,362].

Combinatorial pharmacology can turn rapid state shifts into stable trait change by pairing induction of neuroplasticity with precise excitability control. Ketamine arrests pathologic bursting in the lateral habenula and rebalances prefrontal microcircuits, producing fast antidepressant effects that nevertheless require stabilization to curb relapse [76,364]. Kv7 openers supply a tonic brake on pyramidal neurons via the M-current, lowering burst propensity at the axon initial segment and sharpening gain; diverse chemotypes with translational promise, including QO-83, reduce hyperexcitability and improve cognition in preclinical systems [365,366]. This pharmacodynamic logic supports a sequence in which ketamine initiates synaptogenesis and rebalancing of excitatory/inhibitory (E/I) activity, followed by timed Kv7 augmentation that suppresses rebound bursting and preserves signal fidelity during consolidation [364,367]. Calibration matters, since nonselective Kv7 inhibition by agents like paroxetine can widen excitability and potentially erode benefits if left unchecked [368]. HCN channels offer a complementary lever. Ketamine alters gamma rhythms and inhibits HCN1, while direct HCN modulation produces ketamine-like, sustained antidepressant effects and normalizes midbrain hyperactivity; carefully titrated HCN agents may therefore enhance oscillatory coherence, reduce dysrhythmia, and improve cognitive emotional balance when layered onto ketamine’s plasticity window [76].

A second pairing focuses on limbic damping with DXM-bupropion combined with a GIRK opener. DXM modulates NMDARs and sigma-1 sites, while bupropion supports catecholaminergic tone; together they deliver rapid symptom relief with good tolerability in early clinical work [351,367]. GIRK channels hyperpolarize neurons and raise rheobase, the minimum current required to evoke an action potential, providing a direct counterweight against network hyperactivity implicated across mood and psychosis spectra; potassium channel portfolios already highlight GIRK as a tractable target with cross-diagnostic potential [15,351,365]. Adding a GIRK opener to DXM-bupropion could therefore damp limbic overdrive during plasticity induction, reduce stress reactivity, and steady fronto-limbic coupling, which may translate to fewer lapses and smoother affective control [351,367]. Multimodal trials should test these pairings with target engagement biomarkers, oscillatory readouts, and relapse endpoints, integrating individualized timing maps that align drugs to the patient’s peak plasticity and network state [351,364].

Biomarkers can steer both timing and dose in combination strategies by indexing plasticity readiness and network stability. Three readouts are particularly actionable. First, the EEG spectral slope, a proxy for excitation to inhibition balance, flattens with cortical disinhibition and steepens as inhibition strengthens; tracking slope before and after induction can gate consolidation inputs and prevent overshoot during vulnerable windows [369,370]. Second, rostral anterior cingulate cortex (rACC) theta power is a robust prognostic marker. Higher baseline rACC theta predicts greater symptom improvement across modalities and differentiates responders, enabling dose titration and early switching when trajectories look suboptimal [343,370]. Third, fMRI connectivity within prefrontal limbic circuits forecasts treatment response. Dorsolateral prefrontal cortex (DLPFC)-to-subgenual anterior cingulate cortex (sgACC) and broader salience network coupling stratify rTMS outcomes, while default mode and cingulo frontal patterns predict remission to medication and can guide selection between CBT and pharmacotherapy [370,371]. Multimodal frameworks that combine EEG and fMRI outperform clinical features alone and support precision sequencing in prospective designs [214,372,373].

An adaptive workflow follows a sense decide act loop. Establish a biomarker baseline. Induce plasticity. Recheck EEG slope and rACC theta within hours to map consolidation timing. Adjust neuromodulation targets from fMRI connectivity and iterate dosing or modality accordingly. Closed loop principles, validated in other disorders using electrophysiological control signals, can be translated to psychiatry to deliver responsive, biomarker driven care that prioritizes durability and cognitive emotional balance [343,369] (Table 3).

### 5.3. AMPA-Facilitating Add-Ons

AMPAR facilitating add-ons are discussed here not to restate the ketamine cascade, but to highlight how modest enhancement of AMPAR throughput may support combination strategies after the initial plasticity window. Across pharmacologic and device-based interventions, calibrated AMPAR facilitation may help sustain prefrontal drive, preserve signal-to-noise, and extend remission without excessive excitatory load [380,381,382].

Adjuncts that subtly raise AMPA throughput while engaging monoaminergic tone are gaining clinical traction. Brexpiprazole, a partial dopamine D_2_ and 5-hydroxytryptamine 1A (5-HT_1A_) agonist with 5-hydroxytryptamine 2A (5-HT_2A_) antagonism, facilitates AMPAR-mediated transmission in medial prefrontal cortex through a dopamine D_1_-dependent cascade, particularly when combined with selective serotonin reuptake inhibitors (SSRIs) such as escitalopram [384]. This profile suggests that AMPAR facilitation may serve as a shared downstream gateway through which adjunctive treatments support more durable network reweighting [385]. Because AMPA activation in prefrontal circuits can secondarily recruit dorsal raphe serotonergic output, brexpiprazole may amplify this cortico-monoaminergic loop and improve mood and cognition without excessive excitatory load [384].

Vortioxetine offers a complementary route. Its multimodal serotonergic actions reshape cortical information flow and acutely boost expression of plasticity related genes tied to glutamatergic signaling in frontal cortex, consistent with an AMPA enhancing mechanism distinct from classic SSRIs and temporally separable from ketamine [385]. Convergent frameworks of rapid acting antidepressants place both ketamine and serotonergic agents on a common pathway that culminates in AMPA mediated plasticity and synaptic gain, albeit through different entry points [385]. These overlaps argue for rational pairing or sequencing with AMPA facilitating agents to stabilize prefrontal output, enhance cognitive control, and extend remission while maintaining excitatory safety through targeted dosing and biomarker guided timing [384,385].

**Figure 3:**
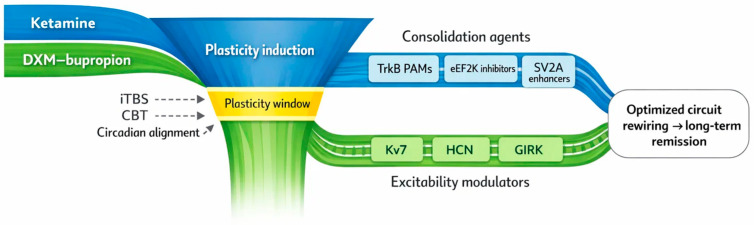
Phase-timed combination strategies to stabilize antidepressant circuit remodeling. This schematic summarizes a staged strategy for converting rapid symptom relief into durable remission. Plasticity inducers such as ketamine or DXM-bupropion open a transient plasticity window during which maladaptive circuit states are most modifiable. Within and shortly after this period, consolidation-focused interventions, including TrkB modulation, eEF2K inhibition, and SV2A enhancement, are positioned to stabilize synaptic strengthening and preserve presynaptic reliability. In parallel, intrinsic excitability modulators targeting Kv7, HCN, and GIRK channels provide gain control, constrain hyperexcitability, and support adaptive oscillatory dynamics. Neuromodulatory and behavioral interventions are depicted as most effective when aligned with this window, thereby reinforcing circuit rewiring and prolonging remission. DXM, dextromethorphan; eEF2K, eukaryotic elongation factor 2 kinase; GIRK, G protein-gated inwardly rectifying potassium channel; HCN, hyperpolarization-activated cyclic nucleotide-gated channel; iTBS, intermittent theta-burst stimulation; Kv7, voltage-gated potassium channel subfamily Q; SV2A, synaptic vesicle glycoprotein 2A; TrkB, tropomyosin receptor kinase B.

Augmentation studies increasingly show that coupling adjuncts to rapid-acting antidepressants improves remission and functional recovery. Network meta-analyses in TRD report higher response and remission with atypical antipsychotic augmentation, with brexpiprazole among the most consistent options [386]. Phase 2 and 3 trials, including Brexpiprazole Efficacy and Safety in Major Depressive Disorder (BLESS) and larger randomized studies at 2 to 3 mg, demonstrate significant symptom and functioning gains with acceptable tolerability, and effects that emerge early and persist across symptom clusters [387]. Real-world switching data echo these benefits, with improvements in depressive symptoms, cognition, and overall functioning after moving to adjunctive brexpiprazole [387]. Mechanistic and preclinical work supports synergy with antidepressants and restoration of plasticity markers, while case series combining brexpiprazole with ketamine or esketamine suggest rapid clinical recovery in complex presentations and motivate controlled multimodal trials [388]. The field now needs longitudinal, biomarker-anchored designs. Stratification by inflammatory load, as in the C-reactive protein (CRP)-guided vortioxetine plus celecoxib protocol, and null findings without stratification underscore the value of precision enrollment and timing analytics for durable remission and cognitive outcomes [49,389] (Figure 3).

## 6. Closing Synthesis and Future Directions

Depression recovery is best understood as a dynamic, multi-phase biological process in which acute relief is encoded, consolidated, and subsequently renormalized across time, with sleep and experience shaping each transition [243]. At its core lie two interacting themes: synaptic plasticity that rewires connectivity, and intrinsic excitability that sets the gain of neuronal ensembles [333]. Stress skews both, degrading cortical and reward circuit function, while rapid-acting and conventional antidepressants restore synaptic strength, spine architecture, and network communication through metaplastic and homeostatic programs that prime future adaptation rather than a single static endpoint [33,390]. Convergent molecular hubs link these levels, notably BDNF–TrkB signaling and PI3K–Akt–mTOR pathways, as well as synaptic organizers such as neurexins that stabilize sustained benefit after agents like ketamine or psilocybin [391]. Bioenergetic resilience and mitochondria further tune plasticity capacity, connecting cellular metabolism to circuit repair and behavior [179]. Translational work shows that macro- and microstructural brain changes track with synaptic remodeling, and that intrinsic network connectivity can forecast remission, underscoring the need for integrated biomarkers that bridge molecules, circuits, and symptoms [179,392]. This section proceeds as follows: a unified model of recovery that couples plasticity with excitability, candidate biomarkers across scales, principles for trial design that assay plasticity readiness, key research gaps, and a future outlook that prioritizes durable, mechanism-anchored interventions [336].

Recovery from depression can be framed as an integrated control problem in which synaptic plasticity writes the map and intrinsic excitability sets the compass. Plasticity induction allocates change to specific synapses through activity-dependent mechanisms, while consolidation stabilizes these changes via molecular programs such as synaptic tagging and capture, coordinated receptor trafficking, and mTOR or CREB signaling that secure long-term efficacy at the appropriate connections [393,394]. In parallel, intrinsic excitability retunes the gain of neuronal ensembles through ion channel modulation, neuromodulatory tone, and inhibitory plasticity, keeping network dynamics within a regime that is both responsive and robust. This pairing allows plasticity to sculpt adaptive connectivity and excitability to ensure stability and precision during information flow and decision making [76,395].

Circuit-level observations make the logic concrete. Coupling between hippocampus and prefrontal cortex depends on long-term potentiation and long-term depression working in concert with oscillatory synchrony under the influence of serotonin, dopamine, and other neuromodulators, and this coupling falters in depression with measurable consequences for cognition and affect [393,396]. In the amygdala, neuroinflammatory states raise glutamatergic drive and intrinsic excitability, biasing engram formation toward threat; targeted disinhibition or serotonergic regulation can rebalance excitation and inhibition and restore controlled retrieval of emotional memories [257,397]. Amygdala activity can also reset the dynamic range of hippocampal plasticity, exemplifying bidirectional regulation across nodes that jointly tune connectivity and gain [398]. At the systems scale, deficits in hippocampal and prefrontal plasticity seen in susceptible strains are reversible when interventions reset both synaptic strength and excitability, whether via pharmacology, experience-dependent enrichment, electroconvulsive therapy, or molecular levers such as SIRT1 that couple intrinsic firing properties to synaptic throughput [12,349,399].

Taken together, therapeutic recovery emerges when connection strength and control are restored simultaneously. Plasticity sets where the network can go; excitability decides how confidently and safely it gets there. Durable remission therefore requires coordinated strategies that induce and consolidate the right synapses while tuning gain to preserve accuracy and resilience across circuits [400].

A multimodal biomarker strategy may eventually connect molecular events at synapses to mesoscopic network dynamics and daily behavior, but its clinical utility remains exploratory. At present, these measures should be interpreted as candidate tools for hypothesis testing, not as validated instruments for treatment selection. SV2A PET may provide a synaptic-tier readout by indexing presynaptic terminal density, but its use for antidepressant optimization remains investigational and requires prospective validation. Yet synaptic density alone does not fully predict functional organization. Combined SV2A PET and resting-state fMRI demonstrate regional network changes that are only partly explained by density, arguing for integrated models rather than single-modality thresholds. Magnetic resonance spectroscopy then quantifies excitatory and inhibitory tone. Glx and GABA levels, particularly in anterior cingulate and prefrontal cortex, correlate with resting connectivity and default-mode interactions, while trimodal PET–MR–EEG indicates that inhibitory processes strongly constrain canonical networks, refining interpretation of E to I balance in vivo. Importantly, unimodal MRS shows inconsistent prognostic utility, reinforcing the need for multimodal designs and larger samples [401,402].

At the network scale, resting-state fMRI yields robust predictors of treatment outcome, including strengthened frontoparietal integration and reliable default-mode suppression; time-resolved metrics such as dwell time in coactivation states forecast early response and capture consolidation dynamics. Electrophysiology closes the temporal gap. Resting EEG topology differentiates responders, while spectral slope and rostral anterior cingulate theta index cortical excitability and plasticity readiness, offering rapid, repeatable assays that complement imaging-derived connectivity maps. Actigraphy supplies the behavioral layer, quantifying circadian alignment and sleep regularity that gate synaptic renormalization and stabilize network states across days [403,404].

**Figure 4:**
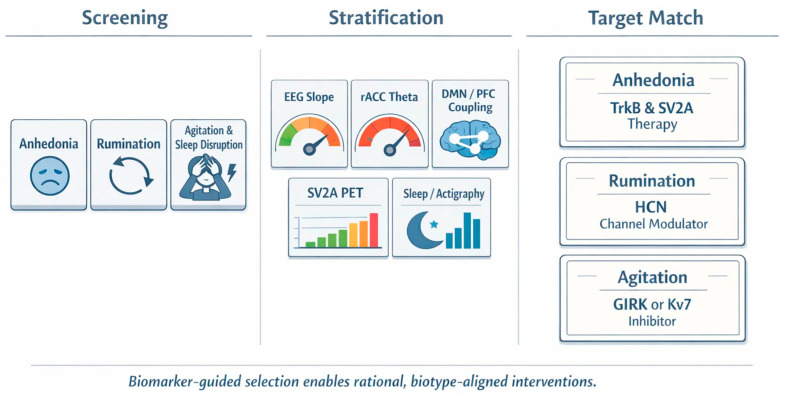
Biotype-guided treatment roadmap for precision antidepressant targeting. The schematic links clinical screening phenotypes—anhedonia, rumination, and agitation/sleep disruption—to biomarker-informed stratification using EEG slope, rACC activity, DMN/PFC coupling, SV2A PET, and sleep/actigraphy readouts. These profiles guide target matching toward TrkB/SV2A-based plasticity support, HCN modulation, or GIRK/Kv7-directed gain control, illustrating how biomarker-guided selection may enable rational, biotype-aligned antidepressant interventions. DMN, default mode network; EEG, electroencephalography; GIRK, G protein-gated inwardly rectifying potassium channel; HCN, hyperpolarization-activated cyclic nucleotide-gated channel; Kv7, voltage-gated potassium channel subfamily Q; PET, positron emission tomography; PFC, prefrontal cortex; rACC, rostral anterior cingulate cortex; SV2A, synaptic vesicle glycoprotein 2A; TrkB, tropomyosin receptor kinase B.

Together, these measures outline a candidate research pipeline rather than a clinically established workflow. SV2A PET, MRS, rs-fMRI, EEG, and actigraphy may help test phase-specific hypotheses, but their ability to guide antidepressant timing, dosing, or switching has not yet been validated in routine care. Actigraphy verifies maintenance by demonstrating entrained rhythms and stable behavior. Multimodal fusion may eventually support individualized signatures, but current evidence does not yet justify real-time clinical course correction or routine biomarker-guided antidepressant switching [405,406] (Figure 4).

Future trials could test whether transient plasticity windows can be clinically leveraged by pairing pharmacological induction with behavioral or neuromodulatory consolidation. Evidence that recovery speed reflects a plasticity by context interplay suggests timing is not a luxury but a mechanism, with interventions succeeding when behavioral input arrives during peak metaplastic readiness rather than after it has waned [398]. Protocols can prespecify window-locked pairings: an inducer to open the gate, followed within hours to days by consolidation tactics such as structured psychotherapy modules, targeted neuromodulation, or sleep and circadian alignment to stabilize network reconfiguration [398]. Multimodal biomarkers embedded at repeated intervals, including circuit scores, EEG indices, and molecular panels, can verify that the window was captured rather than merely assumed [407,408].

Future stratification studies should test whether biologically anchored phenotypes can improve drug-device pairing beyond standard clinical subtypes. High-excitability profiles, indexed by frontolimbic hyperconnectivity or rACC theta, may benefit from consolidation with inhibitory-biased neuromodulation and sleep regularization after a pharmacological primer. Low-plasticity profiles, identified by network inefficiency or proteomic signatures of impaired neurotrophic signaling, may require stronger inducers and cognitive scaffolding to capture newly available synapses. Pharmacogenomic tools add a complementary layer for medication selection, with multiple blinded trials and meta-analyses showing modest yet reliable gains in response and remission, particularly when switching to genetically congruent agents, thereby enriching strata and reducing futile exposure [409,410].

Designs should be adaptive and explicitly longitudinal. SMART frameworks enable data-driven sequencing and timing choices using repeated outcomes and biomarkers to adjust dose, interval, and consolidation intensity in real time [411]. Extensions of 2-in-1 designs allow early selection of responsive biomarker subpopulations while preserving confirmatory power, improving efficiency without sacrificing rigor. Deep learning models that fuse clinical, genetic, neuroimaging, and EEG features can drive interim decision rules, while smartphone-based digital phenotypes provide high-frequency behavioral readouts to refine window placement between visits. Together, these designs could help test timing and patient-selection hypotheses, but they should not be presented as evidence that precision network therapeutics are already clinically reproducible [243,412]. The same framework should also be used to capture negative findings. Failed enrichment strategies, null biomarker effects, and poorly tolerated combinations are essential for defining the boundary conditions of the ICM model (Table 4).

Future work should test, rather than assume, whether drug, device, and behavioral combinations can convert short-lived plasticity into durable clinical benefit. Ketamine paired with intermittent theta burst stimulation is a strong candidate: intravenous dosing opens a brief plasticity gate, while iTBS can steer circuit-specific consolidation to prolong benefit. Signals from rTMS combined with pharmacotherapy on depressive symptoms and sleep quality, along with dose and timing sensitivity of ketamine, motivate prospective evaluation of order, spacing, and intensity. Retrospective and case-based reports of combined TMS and ketamine in resistant depression suggest feasibility, durability, and tolerability at higher stimulation intensities, warranting controlled protocols with standardized parameters. Parallel proofs of concept should examine DXM or related NMDAR modulators coupled to sleep and circadian realignment, using structured schedules or orexin receptor antagonists to stabilize overnight synaptic renormalization and translate acute gains into durable change [429,430].

Trials must be small, fast, and mechanistically anchored. Biomarker batteries should verify the hypothesized interaction: frontolimbic connectivity on functional neuroimaging to index guided consolidation, midline theta or spectral slope to quantify excitability set points, and magnetoencephalography or EEG signatures sensitive to drug and stimulation synergies. Designs should predefine response patterns that trigger within-subject adjustments of dose and intersession interval, converting feasibility pilots into learning engines that refine protocols in real time [431,432].

Translation barriers require explicit mitigation. Preclinical rTMS often uses antidepressant-sensitive strains and stimulation geometries that do not mirror human focality. Species differences in oscillatory markers complicate selection of target frequencies and endpoints. Drug kinetics in animals rarely match human infusion profiles, shifting the induction window relative to stimulation. Mechanistic pilots should harmonize dosing, oscillatory targets, and timing across species, and replicate in treatment-resistant models to better reflect clinical heterogeneity [431,433].

Long-term plasticity enhancement also carries risks that should be built into development programs. Sustained TrkB activation raises theoretical proliferative concerns, but the broader issue is uncontrolled network remodeling. Excessive synaptic strengthening, prolonged translational disinhibition, or poorly calibrated excitability modulation could promote maladaptive plasticity, excitotoxic stress, epileptiform activity, or aberrant stabilization of pathological circuit states. These risks argue for intermittent rather than continuous exposure, circuit-selective delivery, dose ceilings, and longitudinal monitoring with EEG, fMRI, PET, and behavioral readouts. The therapeutic goal is therefore bounded plasticity: strong enough to enable recovery, but constrained enough to preserve network stability [49,434,435].

Equally important is a longitudinal biomarker scaffold that ties molecular engagement to network repair and daily function. Neuroimaging predictors show potential but vary across sites; durable utility will require harmonized protocols, repeated measures, and fusion with electrophysiology that reads cortical gain in real time. Digital phenotyping can supply high-frequency measures of sleep, mobility, and social rhythm, closing the loop between laboratory signals and lived behavior. A practical stack would pair PET or MRS for target engagement, fMRI for evolving connectivity, EEG for excitability dynamics, and smartphones for circadian alignment on a shared temporal axis [436,437].

Computational advances can integrate these layers. Deep graph learning across EEG and fMRI already forecasts treatment response and reveals network signatures yet needs larger prospective datasets and strict controls to prevent data leakage and inflated accuracy (1,15). Next-generation models should simulate circuit-level drug effects, link pharmacokinetics to network dynamics, and output dose–timing schedules that can be pre-registered and tested in confirmatory trials [438,439].

This structure provides the mechanistic unification of the review. Rather than grouping targets by molecular family alone, the ICM model assigns each mechanism a temporal role: AMPAR throughput opens the window, TrkB and eEF2K stabilize the rewrite, SV2A protects presynaptic fidelity, and Kv7, HCN, and GIRK channels tune the excitability set point that determines durability [33,243].

Within this model, next-generation agents are network reprogrammers, not mere neurotransmitter correctors. They will target plasticity hubs and adhesion systems that determine where connections form, while tuning intrinsic excitability to decide how precisely they operate in context. Personalization remains a future objective rather than an established clinical outcome. Multi-point interventions may eventually pair a plasticity inducer with neuromodulation and behavioral entrainment, but this approach requires controlled trials with predefined biomarkers, safety endpoints, and replication across sites. Computational tools that integrate fMRI and EEG already recover predictive signatures of response and can propose dose and timing schedules aligned to individual network states [14,37,181]. In sum, the ICM framework organizes antidepressant discovery around three testable phases: induction, consolidation, and maintenance. Its clinical value now depends on prospective validation, safety testing, and reproducible biomarkers [243].

These translational constraints should temper the interpretation of all candidate maintenance and consolidation strategies. TrkB PAMs, eEF2K inhibitors, SV2A modulators, and Kv7 openers each face a different bottleneck: uncertain target specificity and BBB delivery for TrkB agents, lack of clinical-grade compounds and off-target kinase risk for eEF2K inhibition, repurposing uncertainty and behavioral tolerability concerns for SV2A ligands, and discontinued or tolerability-limited clinical experience for earlier Kv7 openers. In addition, long-term modulation of neuroplasticity pathways may not always be beneficial. Excessive or poorly timed intervention could stabilize maladaptive circuits, alter excitation inhibition balance, or produce network states that are durable but not adaptive. Future studies should therefore prioritize target engagement, CNS exposure, reproducibility, long-term safety, and phenotype-specific benefit before these approaches are framed as clinically actionable antidepressant strategies. Limitations of the proposed framework. The ICM framework is intended as a narrative synthesis rather than a validated clinical algorithm. Ketamine and esketamine provide the strongest clinical anchor, whereas TrkB modulators, eEF2K inhibitors, SV2A enhancers, and ion-channel strategies remain at earlier stages of translation. Biomarkers such as SV2A PET, MRS, EEG, fMRI, and actigraphy may support mechanistic studies and trial enrichment, but they are not yet validated tools for routine patient stratification. Long-term modulation of glutamatergic plasticity, TrkB signaling, translational control, SV2A pathways, or intrinsic excitability also requires careful safety evaluation. Long-term modulation of glutamatergic plasticity, TrkB signaling, translational control, SV2A pathways, or intrinsic excitability requires careful safety evaluation. Key concerns include maladaptive plasticity, excitability imbalance, seizure-threshold changes, off-target signaling, adverse neuropsychiatric effects, abuse liability, and uncertainty related to chronic ketamine or esketamine exposure.

Several boundaries should be kept explicit. Ketamine and esketamine provide the strongest clinical anchor for rapid glutamatergic antidepressant action, whereas TrkB potentiation, eEF2K inhibition, SV2A modulation, and ion-channel strategies remain at different and generally earlier stages of translation. Biomarker discussions should therefore be read as research priorities rather than clinical algorithms. Similarly, the distinction between synaptic plasticity and intrinsic excitability is mechanistic: synaptic plasticity alters connection strength and structure, whereas intrinsic excitability alters input-output gain and firing stability. The ICM framework is intended to synthesize these strands in a balanced narrative review, not to present a fully validated precision-therapeutic model. Accordingly, the figures and tables should be read as organizing tools that compare mechanistic plausibility, translational maturity, and safety constraints, not as evidence that all pathways or targets are equally validated. This caution is especially important for ion-channel targets, where physiological plausibility and rodent behavioral effects should not be conflated with clinically meaningful antidepressant efficacy.

## 7. Conclusions

Effective antidepressant discovery increasingly depends on recognizing that lasting recovery involves synaptic plasticity and intrinsic excitability, while also being shaped by immune, glial, metabolic, endocrine, developmental, and sex-specific mechanisms. Across this review, antidepressant action is framed as a staged process: induction initiates synaptic change, consolidation stabilizes it, and maintenance preserves circuit function. In this framework, monoamines may contribute to symptomatic improvement, but durable remission requires reorganization of the neural networks that encode mood, cognition, and stress responsiveness. Antidepressant action is therefore best understood not as simple neurotransmitter replacement, but as coordinated circuit repair. This framing is not meant to imply a single deterministic pathway. Rather, circuit repair emerges from interacting neuronal, glial, immune, metabolic, and endocrine processes that vary across patients and illness stages.

This model differs from earlier neuroplasticity-centered frameworks by assigning intrinsic excitability a defined mechanistic role rather than treating it as a secondary consequence of synaptic change. Synaptic plasticity explains how circuits are remodeled, whereas excitability tuning explains how remodeled circuits are stabilized, filtered, and protected from relapse-prone network drift. These translational applications should be interpreted cautiously. Biomarker-guided stratification, SV2A PET monitoring, and phase-specific circuit interventions remain promising research directions rather than validated clinical strategies.

Synaptic plasticity explains how circuits are remodeled, whereas excitability tuning explains how remodeled circuits are stabilized, filtered, and protected from relapse-prone network drift. Equally, long-term interventions must avoid overconsolidating maladaptive network states, increasing excitability beyond safe limits, or destabilizing excitation inhibition balance. A balanced interpretation also requires negative evidence: failed NMDA-modulator programs, inconsistent ketamine biomarker findings, unresolved TrkB reproducibility issues, and unvalidated SV2A PET applications define the current limits of translation. Accordingly, the ICM framework should be interpreted as a hypothesis-generating synthesis rather than a systematic ranking of evidence strength across targets, biomarkers, or interventions. For ion-channel targets in particular, the present evidence is best viewed as a graded continuum, moving from established excitability physiology to preclinical antidepressant-like effects and only limited clinical validation. Looking ahead, the next generation of antidepressants will not simply promote plasticity; they will coordinate plasticity with excitability control so that rapid circuit rewiring becomes stable, measurable, and clinically durable. Clinical translation remains constrained by limited durability, unclear biomarker validity, relapse after response, adverse-effect burden, and the absence of reproducible patient stratification.

## Figures and Tables

**Table 1:** This table separates mechanistic plausibility from clinical maturity. Established clinical evidence, failed or discontinued development paths, preclinical-only targets, biomarker readiness, and major safety barriers are distinguished to avoid presenting broad therapeutic categories as equally validated.

Molecular Target or Lever	Mechanism in Synaptic Plasticity	Representative Preclinical Evidence	Clinical Translation Status	Evidence Maturity	Associated Biomarkers or Assays	Major Safety Considerations	References
Glutamate drivers: NMDAR antagonism, AMPAR facilitation, partial NMDAR modulation	Disinhibition and glutamate surge increase AMPAR throughput, opening a transient plasticity window that recruits BDNF-mTORC1 remodeling programs.	Ketamine-like paradigms restore spine density and excitation-inhibition balance in stress models, aligning behavior with synaptic strengthening.	Ketamine and esketamine are clinically validated for rapid response; partial NMDAR modulators include failed development programs and require stronger replication.	Approved clinical use; failed or mixed clinical translation	MRS Glx or GABA; EEG or MEG spectra; rs-fMRI connectivity; peripheral BDNF signaling panels.	Dissociation, BP elevation, abuse liability, sedation, rebound risk, and excessive excitation in susceptible circuits.	[167,168,169]
TrkB: BDNF-TrkB agonists or positive allosteric modulators	TrkB acts as a consolidation gate, stabilizing nascent spines and coupling local synaptic events to transcriptional persistence.	TrkB PAMs and 7,8-DHF analogs prolong ketamine-linked behavioral signatures and support spine stabilization.	Experimental consolidation target; proposed TrkB agonists and PAMs remain limited by target specificity, reproducibility, BBB delivery, and sparse human data.	Investigational adjunct	BDNF dynamics; pTrkB, pERK, or pAkt readouts; PET or fMRI integration; TMS-EEG plasticity.	Seizure-threshold shifts, off-target kinase effects, proliferative signaling concerns, and need for controlled brain exposure.	[170,171,172]
eEF2K: elongation checkpoint and translational disinhibition	eEF2K inhibition reduces eEF2 phosphorylation, lifting a local translation brake that supports maturation of potentiated synapses.	Pharmacologic or genetic eEF2K reduction enhances BDNF translation, AMPAR transmission, and antidepressant-like behavior.	Preclinical target only; no clinical-grade antidepressant candidate has established brain penetration, selectivity, chronic safety, or human efficacy.	Preclinical only	p-eEF2 to total eEF2 ratios; synaptic proteomics; immediate early genes; LTP and spine maturation.	Unintended global translation, metabolic stress, seizure-related interactions, and off-target kinase effects require cautious dosing.	[146,148,153]
SV2A: vesicle cycling stabilizer and synaptic density anchor	SV2A stabilizes vesicle priming, calcium-coupled release, and terminal integrity after postsynaptic remodeling.	SV2A modulation preserves synaptic structure in stress and neurodegeneration models; levetiracetam-class ligands support circuit resilience.	Repurposing is feasible through approved ligands, but antidepressant efficacy and SV2A PET-guided optimization remain unvalidated.	Investigational adjunct; research biomarker	SV2A PET ligands; longitudinal PET-symptom coupling; CSF or extracellular-vesicle synaptic protein panels.	Irritability, mood lability, sedation, fatigue, dosing-window uncertainty, and long-term tolerability concerns.	[173,174,175]

7,8-DHF, 7,8-dihydroxyflavone; AMPAR, α-amino-3-hydroxy-5-methyl-4-isoxazolepropionic acid receptor; BDNF, brain-derived neurotrophic factor; CSF, cerebrospinal fluid; eEF2K, eukaryotic elongation factor 2 kinase; fMRI, functional magnetic resonance imaging; Glx, glutamate plus glutamine; LTP, long-term potentiation; MEG, magnetoencephalography; mTORC1, mechanistic target of rapamycin complex 1; MRS, magnetic resonance spectroscopy; NMDAR, N-methyl-D-aspartate receptor; PAM, positive allosteric modulator; PET, positron emission tomography; pAkt, phosphorylated protein kinase B; pERK, phosphorylated extracellular signal-regulated kinase; pTrkB, phosphorylated tropomyosin receptor kinase B; rs-fMRI, resting-state functional magnetic resonance imaging; SV2A, synaptic vesicle glycoprotein 2A; TMS-EEG, transcranial magnetic stimulation electroencephalography; TrkB, tropomyosin receptor kinase B.

**Table 2:** Phenotype-linked intrinsic excitability targets for biomarker-guided antidepressant translation and dosing. This table compares Kv7, HCN, and GIRK targets across mechanism, phenotype alignment, preclinical support, clinical maturity, and biomarker strategy. The emphasis is on concise gain-control logic: reducing maladaptive bursting, tuning dendritic resonance, or restoring inhibitory tone while using network and arousal readouts to guide dosing and sequencing.

Channel Target/Compartment	Representative Modulators	Mechanistic Lever	Phenotype-Linked Profile	Preclinical Highlights	Clinical Translation	Biomarker Strategy	References
Kv7 (KCNQ2–5) Soma, AIS, proximal dendrite	Retigabine or ezogabine; next-generation Kv7.2/7.3-biased agonists.	M-current enhancement raises spike threshold, reduces bursting, and stabilizes excitability after induction.	Anxiety, affective lability, hyperarousal, and relapse-prone hyperexcitability.	Openers dampen cortical and limbic hyperexcitability, normalize ensembles, and reduce anxiety-like behavior.	Human retigabine experience supports target engagement, but tolerability limits psychiatric translation.	EEG spectral slope, PFC-limbic coupling, sleep and actigraphy readouts.	[245,328,329]
HCN (Ih; HCN1-enriched) Distal dendritic shaft	ZD7288 tools; ivabradine; tDCS or DBS affecting Ih-sensitive circuits.	Ih tuning reshapes dendritic resonance, temporal integration, default-mode drive, and oscillatory coherence.	Rumination, cognitive inertia, slowed affective updating, and maladaptive DMN engagement.	Partial HCN reduction normalizes DMN-like hyperconnectivity and reduces rumination-like behavior.	Selective agents remain scarce; translation favors phenotype-defined neuromodulation and biomarker guidance.	DMN connectivity, theta coherence, slow-wave power, and EEG slope.	[271,330,331]
GIRK (Kir3.x) Soma and dendrites	ML297, ML29, GABA_B and 5-HT1A pathways engaging GIRK.	G-protein-coupled potassium conductance restores inhibitory tone, lowers input resistance, and reduces bursting.	Agitation, irritability, sleep instability, and stress-evoked limbic overdrive.	Activation reduces circuit noise, avoidance behavior, anxiety-like responses, and sleep disruption.	Human translation remains early; subtype selectivity and brain exposure are limiting.	EEG slope, arousal metrics, limbic-prefrontal coupling, agitation and sleep endpoints.	[302,304,314]

AIS, axon initial segment; DBS, deep brain stimulation; DMN, default mode network; EEG, electroencephalography; fMRI, functional magnetic resonance imaging; GABA_B, γ-aminobutyric acid type B receptor; GIRK, G protein-gated inwardly rectifying potassium channel; HCN, hyperpolarization-activated cyclic nucleotide-gated channel; Ih, hyperpolarization-activated current; KCNQ2–5, potassium voltage-gated channel subfamily Q member 2–5; Kv7, voltage-gated potassium channel subfamily Q; PFC, prefrontal cortex; tDCS, transcranial direct current stimulation; 5-HT1A, 5-hydroxytryptamine receptor 1A.

**Table 3:** Phase-timed combination strategies for durable antidepressant circuit stabilization and remission. This table summarizes ICM-based pairings that link rapid induction, consolidation support, and maintenance stabilization. Each strategy is aligned with likely phenotype fit, monitoring readouts, relapse-prevention logic, and implementation cautions. The examples are conceptual and should guide hypothesis-driven trials rather than imply established clinical protocols.

ICM Strategy	Induction	Consolidation	Maintenance	Clinical Fit	Monitoring	Relapse Logic	Implementation Notes	References
Ketamine plus TrkB PAM plus iTBS	Ketamine or esketamine opens a short plasticity window and reduces symptoms quickly.	TrkB PAMs bias BDNF signaling toward spine stabilization and synaptic persistence.	iTBS reinforces adaptive network reweighting during the consolidation window.	Best for anhedonia, cognitive rigidity, and stress-reactive depression.	EEG slope, rACC theta, rs-fMRI coupling, and symptom dynamics.	Combines molecular consolidation with circuit training to extend remission.	Time iTBS within hours to days; monitor dissociation, BP, and anxiety.	[374,375,376]
DXM-bupropion plus eEF2K inhibition	DXM-bupropion provides multimodal NMDA, sigma-1, and catecholaminergic induction signals.	eEF2K inhibition releases translational braking and supports protein-dependent stabilization.	Behavioral activation strengthens task-coupled learning during consolidation.	May fit low drive, rumination, and motivational slowing.	EEG slope, task-evoked ERP, and cognitive-control network connectivity.	Translation-centered consolidation may improve durability beyond symptomatic induction.	Review interactions, screen seizure risk, and favor daytime dosing.	[62,156,377]
Esketamine plus Kv7 opener	Intranasal esketamine triggers rapid symptom reduction and synaptogenic signaling.	Gain stabilization limits post-induction hyperexcitability that may destabilize remodeling.	Kv7 opening lowers firing gain and suppresses relapse-prone bursting.	Best for agitation, hyperarousal, affective lability, and stress reactivity.	EEG slope, sleep or actigraphy, rs-fMRI coupling, and arousal metrics.	Directly counters rebound excitation and supports post-induction network stability.	Use cautious low-dose maintenance; monitor sedation, dizziness, and agent-specific AEs.	[262,378,379]
Ketamine plus timed rapamycin adjunct	Ketamine initiates rapid plasticity and early symptomatic relief.	Timed rapamycin may shape immune-metabolic conditions supporting consolidation.	Antidepressant backbone and psychotherapy support maintenance after induction.	May suit relapse-prone courses or inflammatory signatures.	Inflammatory markers, EEG slope, and relapse timing trajectory.	Modifies the consolidation environment to protect newly remodeled networks.	Screen infection risk, manage immunosuppression, and coordinate specialist oversight.	[48,243,380]
Ketamine or esketamine plus lithium maintenance	Ketamine or esketamine produces rapid acute symptom reduction.	Sleep alignment and learning-based interventions support early consolidation.	Lithium stabilizes homeostatic set points and reduces recurrence risk.	Fits recurrent, high-risk depression with mood instability.	Relapse history, sleep regularity, and supportive EEG slope measures.	Maintenance stabilization reduces drift toward maladaptive attractor states.	Monitor renal and thyroid function, hydration, interactions, and toxicity signals.	[243,381,382]
Rapid inducer plus SV2A ligand stabilization	Ketamine, esketamine, or DXM-bupropion initiates plasticity.	Rehabilitation or psychotherapy supports activity-dependent consolidation.	SV2A ligands may stabilize vesicle cycling and release fidelity.	Exploratory option for irritability, agitation, or circuit noise.	SV2A PET, EEG slope, and rs-fMRI network stability.	Presynaptic stabilization may preserve strengthened synapses and reduce release variability.	Use within research frameworks; monitor irritability, sedation, and mood AEs.	[175,220,383]

AEs, adverse events; BDNF, brain-derived neurotrophic factor; BP, blood pressure; DXM, dextromethorphan; eEF2K, eukaryotic elongation factor 2 kinase; ERP, event-related potential; ICM, induction–consolidation–maintenance; iTBS, intermittent theta-burst stimulation; Kv7, voltage-gated potassium channel subfamily Q; NMDA, N-methyl-D-aspartate; PAM, positive allosteric modulator; PET, positron emission tomography; rACC, rostral anterior cingulate cortex; rs-fMRI, resting-state functional magnetic resonance imaging; SV2A, synaptic vesicle glycoprotein 2A; TrkB, tropomyosin receptor kinase B.

**Table 4:** Research gaps and biomarker-guided experimental paths for durable antidepressant plasticity. This table summarizes unresolved questions across postsynaptic consolidation, presynaptic stabilization, intrinsic gain control, drug-device timing, biomarker stratification, and relapse biology. Each domain is paired with concise experimental approaches, candidate readouts, readiness milestones, and safety priorities to guide cautious translation toward durable antidepressant benefit. The table should be read as a research agenda, not as a map of clinically validated precision therapeutics.

Target or Domain	Key Unanswered Question	Why It Matters	Proposed Experimental Approach	Biomarkers or Readouts	Readiness Milestone	Safety Focus	References
TrkB consolidation	When is TrkB potentiation adaptive versus maladaptive?	Determines spine durability, regional stability, and timing with rapid inducers.	Map timing after ketamine or esketamine with TrkB PAMs and learning tasks.	EEG slope; rACC theta; rs-fMRI coupling; flexibility tasks.	Define timing, dose, responder phenotype, and target engagement.	Monitor hypomania, sleep disruption, overstimulation, and regional overactivation.	[348,375,413]
eEF2K checkpoint	Which cells require minimal translation release for persistence?	Prevents broad protein synthesis while preserving consolidation benefit.	Use spine-resolved proteomics with selective tools and DXM-bupropion-like induction.	EEG or ERP plasticity; synaptic proteins; control-network rs-fMRI.	Show CNS penetration, dose-response, biomarkers, and pairing logic.	Assess kinase off-targets, seizure threshold, and drug interactions.	[153,156,414]
SV2A stabilization	Does SV2A modulation prolong remission through vesicle cycling?	Tests whether presynaptic maintenance extends rapid-response durability.	Add SV2A ligands after induction with PET and relapse tracking.	SV2A PET; EEG variability; rs-fMRI stability; actigraphy relapse markers.	Link SV2A PET change to symptom durability and biotype.	Track irritability, mood lability, cognition, and dosing tolerability.	[216,218,415]
Kv7 anti-burst control	Can Kv7 opening prevent rebound without blocking plasticity?	Balances post-induction stability against excessive suppression of consolidation.	Compare delayed versus concurrent Kv7 opening after rapid induction.	EEG slope; sleep stability; arousal metrics; PFC-limbic rs-fMRI.	Identify timing that preserves response and extends time-to-relapse.	Monitor sedation, dizziness, urinary effects, vision, and cognition.	[262,270,416]
HCN resonance control	Which HCN direction and region treat rumination-dominant biotypes?	HCN effects are circuit-specific; wrong targeting may worsen cognition.	Pair neuromodulation with Ih tuning in imaging-guided crossover designs.	DMN rs-fMRI; theta coherence; slow-wave power; rumination tasks.	Validate brain-penetrant modulators or device-based circuit proxies.	Watch bradycardia, processing speed, attention, and sleep architecture.	[290,417,418]
GIRK inhibitory tone	Can GIRK activation reduce agitation without affective blunting?	Targets high-arousal relapse pathways through GPCR-coupled inhibitory reserve.	Develop subtype-biased GIRK modulators and test sleep-first endpoints.	EEG slope; arousal metrics; polysomnography; limbic-prefrontal rs-fMRI.	Demonstrate brain penetration, target physiology, and symptom-specific benefit.	Assess sedation, dizziness, sedative interactions, and motivational blunting.	[320,323,419]
Drug-device timing	When should iTBS, ECT, or tDCS follow induction?	Defines scalable protocols that convert transient plasticity into durability.	Vary stimulation onset after dosing using closed-loop EEG triggers.	EEG slope; theta markers; symptom time-series; rs-fMRI coupling.	Establish timing algorithms, workflow feasibility, and responder enrichment.	Monitor seizure risk, autonomic effects, dissociation, and anxiety.	[420,421,422]
Biomarker biotypes	Which panel assigns patients to consolidation or gain-control adjuncts?	Reduces heterogeneity and enables physiology-guided antidepressant sequencing.	Pre-register EEG-fMRI strata and assign adjuncts by physiology.	EEG slope; rACC theta; DMN coupling; SV2A PET subset.	Prospective evidence that stratification improves outcomes beyond clinical selection alone.	Limit false stratification, site variability, cost, and patient burden.	[423,424,425]
Durability and relapse biology	What maintains remission months after rapid induction?	Guides maintenance duration and selection after initial response.	Run longitudinal biomarker cohorts with relapse modeling and learning probes.	SV2A PET; EEG drift; rs-fMRI stability; digital relapse data.	Define relapse predictors and stopping rules for maintenance therapy.	Track tolerance, dependence-like patterns, cognition, and chronic safety.	[426,427,428]

CNS, central nervous system; DMN, default mode network; DXM, dextromethorphan; ECT, electroconvulsive therapy; EEG, electroencephalography; eEF2K, eukaryotic elongation factor 2 kinase; ERP, event-related potential; GIRK, G protein-gated inwardly rectifying potassium channel; GPCR, G protein-coupled receptor; HCN, hyperpolarization-activated cyclic nucleotide-gated channel; iTBS, intermittent theta-burst stimulation; Ih, hyperpolarization-activated current; Kv7, voltage-gated potassium channel subfamily Q; PAMs, positive allosteric modulators; PET, positron emission tomography; PFC, prefrontal cortex; rACC, rostral anterior cingulate cortex; rs-fMRI, resting-state functional magnetic resonance imaging; SV2A, synaptic vesicle glycoprotein 2A; tDCS, transcranial direct current stimulation; TrkB, tropomyosin receptor kinase B.

## Data Availability

No new data were created or analyzed in this study.

## Abbreviations

The following abbreviations are used in this manuscript:

2R,6R-HNK	(2R,6R)-hydroxynorketamine
5-HT_1A_	5-hydroxytryptamine receptor 1A
5-HT_2A_	5-hydroxytryptamine 2A
7,8-DHF	7,8-dihydroxyflavone
^11^C	carbon-11
^18^F	fluorine-18
AGN-241751	4-chlorokynurenine [AV-101]
AIS	axon initial segment
AKT	protein kinase B
AMPA	α-amino-3-hydroxy-5-methyl-4-isoxazolepropionic acid
AMPAR	α-amino-3-hydroxy-5-methyl-4-isoxazolepropionic acid receptor
Arc	activity-regulated cytoskeleton-associated protein
A/T/N	amyloid/tau/neurodegeneration
BBB	blood-brain barrier
BDNF	brain-derived neurotrophic factor
BLESS	Brexpiprazole Efficacy and Safety in Major Depressive Disorder
BP	blood pressure
CA1	Cornu Ammonis area 1
CaMKII	calcium/calmodulin-dependent protein kinase II
cAMP	cyclic adenosine monophosphate
CBD	Cannabidiol
CBT	cognitive behavioral therapy
CNBD	cyclic nucleotide–binding domain
CNS	central nervous system
CP-AMPAR	calcium-permeable AMPA receptor
CREB	cyclic adenosine monophosphate response element–binding protein
CRP	C-reactive protein
CSF	cerebrospinal fluid
DBS	deep brain stimulation
DHEAS	dehydroepiandrosterone sulfate
DLPFC	dorsolateral prefrontal cortex
DMN	default mode network
DXM	Dextromethorphan
ECT	electroconvulsive therapy
eEF2	eukaryotic elongation factor 2
eEF2K	eukaryotic elongation factor 2 kinase
E/I	excitation/inhibition
EPSC	excitatory postsynaptic current
EPSP	excitatory postsynaptic potential
ERK	extracellular signal-regulated kinase
ERP	event-related potential
FMRP	fragile X mental retardation protein
GABA	gamma-aminobutyric acid
GABA_B	gamma-aminobutyric acid type B receptor
Gβγ	G protein βγ
GEMINI	Global Evaluation of the Efficacy and Safety of AXS-05 [dextromethorphan–bupropion] in Major Depressive Disorder
GIRK	G protein-gated inwardly rectifying potassium channel
GIRK1	G protein-gated inwardly rectifying potassium channel 1 (Kir3.1)
GIRK2	G protein-gated inwardly rectifying potassium channel 2 (Kir3.2)
GluA2	glutamate ionotropic receptor AMPA type subunit 2
GPCR	G protein-coupled receptor
GTPase	guanosine triphosphatase
HCN	hyperpolarization-activated cyclic nucleotide-gated channel
HCN1	hyperpolarization-activated cyclic nucleotide-gated channel 1
HCN4	hyperpolarization-activated cyclic nucleotide-gated channel 4
ICM	Induction → Consolidation → Maintenance
ICU	intensive care unit
Ih	hyperpolarization-activated current
iTBS	intermittent theta-burst stimulation
Kcnq2	potassium voltage-gated channel subfamily Q member 2
KCNQ2–5	potassium voltage-gated channel subfamily Q member 2–5
Kir2	inwardly rectifying potassium channel subfamily 2
Kir3.x	inwardly rectifying potassium channel subfamily 3
Kv7	voltage-gated potassium channel subfamily Q
Kv7.3	voltage-gated potassium channel subfamily Q member 3
LTP	long-term potentiation
MADRS	Montgomery–Åsberg Depression Rating Scale
MeCP2	methyl-CpG-binding protein 2
mGlu2/3	metabotropic glutamate receptor 2 and 3
MRS	magnetic resonance spectroscopy
mTOR	mechanistic target of rapamycin
mTORC1	mechanistic Target Of Rapamycin Complex 1
NAc	nucleus accumbens
NLRP3	NOD-, LRR-, and pyrin domain–containing protein 3
NMDA	N-methyl-D-aspartate
NMDAR	N-methyl-D-aspartate receptors
NREM	non–rapid eye movement
p75NTR	p75 neurotrophin receptor
PAM	positive allosteric modulator
PET	positron emission tomography
PFC	prefrontal cortex
PK-PD	pharmacokinetic–pharmacodynamics
POC	proof of concept
proBDNF	precursor brain-derived neurotrophic factor
rACC	rostral anterior cingulate cortex
RGS	G protein signaling
rTMS	Repetitive transcranial magnetic stimulation
sgACC	subgenual anterior cingulate cortex
SSRI	selective serotonin reuptake inhibitor
SV2A	synaptic vesicle glycoprotein 2A
TBS	enhanced remission durability
tDCS	transcranial direct current stimulation
TMS	transcranial magnetic stimulation.
TRD	treatment-resistant depression
TRIP8b	tetratricopeptide repeat–containing Rab8b-interacting protein
TrkB	tropomyosin receptor kinase B
TRPV1	transient receptor potential vanilloid 1
VTA	ventral tegmental area
ZD7288	HCN channel blocker compound ZD7288
